# Towards designer organelles by subverting the peroxisomal import pathway

**DOI:** 10.1038/s41467-017-00487-7

**Published:** 2017-09-06

**Authors:** Laura L. Cross, Rupesh Paudyal, Yasuko Kamisugi, Alan Berry, Andrew C. Cuming, Alison Baker, Stuart L. Warriner

**Affiliations:** 1 Astbury Centre for Structural Molecular Biology, University of Leeds, Leeds, LS2 9JT UK; 2 School of Chemistry, University of Leeds, Leeds, LS2 9JT UK; 3 School of Molecular and Cellular Biology, University of Leeds, Leeds, LS2 9JT UK; 4 The Centre for Plant Sciences, University of Leeds, Leeds, LS2 9JT UK; 5 School of Biology, University of Leeds, Leeds, LS2 9JT UK

## Abstract

The development of ‘designer’ organelles could be a key strategy to enable foreign pathways to be efficiently controlled within eukaryotic biotechnology. A fundamental component of any such system will be the implementation of a bespoke protein import pathway that can selectively deliver constituent proteins to the new compartment in the presence of existing endogenous trafficking systems. Here we show that the protein–protein interactions that control the peroxisomal protein import pathway can be manipulated to create a pair of interacting partners that still support protein import in moss cells, but are orthogonal to the naturally occurring pathways. In addition to providing a valuable experimental tool to give new insights into peroxisomal protein import, the variant receptor-signal sequence pair forms the basis of a system in which normal peroxisomal function is downregulated and replaced with an alternative pathway, an essential first step in the creation of a designer organelle.

## Abstract (web summary)

Designer organelles could allow the isolation of synthetic biological pathways from endogenous components of the host cell. Here the authors engineer a peroxisomal protein import pathway orthogonal to the naturally occurring system.

## Introduction

Subcellular compartmentalization is a fundamental process used extensively in eukaryotes to separate potentially incompatible biological reaction pathways and processes, as well as enabling locally high concentrations of key pathway components or the segregation of harmful products. The creation of a bespoke intracellular compartment would be a highly desirable synthetic biology tool, as it would enable non-natural pathways to be isolated from other cellular processes. Such isolation could lead to increased yields of synthetic proteins in biotechnology applications and prevention of adverse effects on existing metabolic pathways, as well as alteration of post-translational modifications of high-value protein products. In order to create such compartments, it will be necessary to create tailored and specific delivery systems by manipulating the extensive cellular protein trafficking networks that deliver cargo selectively to organelles. Here we show that it is possible to remodel the protein–protein interactions that control peroxisomal protein import to create an orthogonal signal-receptor pair that is functional in vivo. This allows switching of the selectivity of protein import to effectively ‘hijack’ the function of the pre-existing peroxisome, simultaneously downregulating import of native peroxisomal proteins — an essential first step towards creating a designer organelle that could exist in parallel with ‘normal’ peroxisomes.

The peroxisome is an ideal starting point for the development of a customizable compartment:^1^ it does not contain a genome, so all proteins are imported from the cytosol, and proteins are imported in a fully folded state through a transient pore, so peroxisomes retain a barrier to the cytosol^2^. The peroxisome has already been identified as a compartment for biotechnological exploitation: non-peroxisomal biosynthetic enzymes can be directed to the yeast peroxisome and still retain function^3–5^, while targeting synthetic pathways to peroxisomes can increase the production of fatty-acid-derived alcohols, alkanes, and olefins by up to 700%^6^.

Protein targeting to peroxisomes depends on recognition of a short peptide signal sequence by a receptor that cycles between the cytosol and the peroxisome. A C-terminal peroxisomal targeting signal 1 (PTS1) is the predominant signal in peroxisomal proteins, and this is recognized by the receptor peroxin 5 (PEX5). PEX5 binds PTS1, escorts the PTS1-containing protein to the peroxisomal membrane, and then (together with peroxisomal membrane proteins) inserts into the membrane, creating a dynamic pore through which the peroxisomal protein is delivered to the organellar lumen^7–9^. PEX5 is recycled back to the cytosol in a process driven by ubiquitination and ATP hydrolysis^10^. PTS1 is not a single motif but rather a family of sequences and, while a C-terminal tripeptide sequence of [S/A]-[K/R]-[L/M] is optimal, a range of non-consensus residues can be tolerated in the correct upstream context^11–13^.

**Fig. 1:**
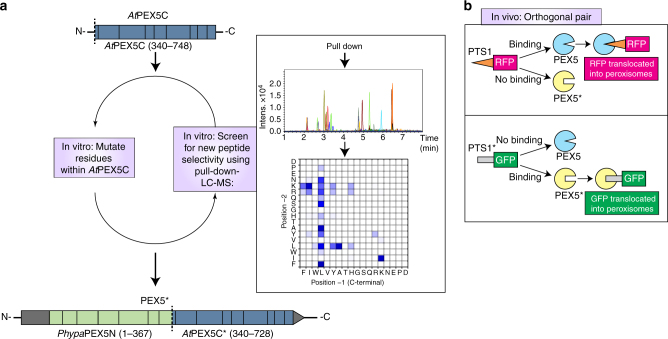
Strategy for the development of an orthogonal PEX5:PTS1-like interaction. **a** The C-terminal domain of *At*PEX5 (*At*PEX5C) was mutated and screened in vitro against a library of peptides to identify binding of non-PTS1 sequences. Once an orthogonal pair of peptide (PTS1*) and protein (PEX5*) was identified, the mutant *At*PEX5C* sequence was then fused to *Phypa*PEX5N to create a hybrid full-length receptor with altered specificity suitable for in vivo experiments in the moss *P. patens (Phypa)*. **b** The intracellular localization of fluorescent proteins appended with either a PTS1 or PTS1* sequence was then used to determine the orthogonality of import in vivo

PEX5 is a modular protein: the C-terminal domain recognizes PTS1 within a funnel-shaped pocket created by 7 α-helical tetratricopeptide repeats (TPRs)^14^, while the N-terminal domain of PEX5, which is natively unstructured^15^, is responsible for the docking, cargo delivery, and recycling functions of the receptor^10^. We hence envisioned that the C-terminal domain of PEX5 could be mutated to produce a variant with orthogonal targeting sequence recognition (PEX5*) without altering the import competence of the variant receptor. The PEX5*-PTS1* pair was discovered by assessing the binding of a range of variants of the C-terminal domain of *Arabidopsis thaliana* PEX5 (*At*PEX5C) with a library of pentapeptides (Fig. 1a). PEX5* shows greatly reduced binding to a representative PTS1 peptide and a ~300-fold increase in affinity for an orthogonal peptide sequence (PTS1*). PTS1* has low affinity for the naturally occurring PEX5 receptor. Following optimization, the C-terminal PEX5* sequence was combined with the N-terminal domain of PEX5 from the moss *Physcomitrella patens* (*Phypa* PEX5N) to create a fully functional PEX5* receptor (Fig. 1a). Concomitant expression of PEX5* with a cargo protein bearing PTS1* results in targeting of the cargo to the peroxisome at the expense of naturally occurring peroxisomal components—a process that was easily visualized in moss cells using fluorescent reporter proteins (Fig. 1b).

## Results

### Assaying for protein-binding peptides

The chemical space that can be covered by all possible variants of the receptor and signal sequence is vast (e.g., combined randomization of the two C-terminal PTS1 residues and just six residues within *At*PEX5C would result in a library size of 2.6 × 10^10^ variants of peptide and protein), which makes the development of an assay for a PEX5*-PTS1* pair problematic. We reduced the dimensionality of this search by designing modifications to the PEX5 component and rapidly surveying which peptide sequences could be bound by each mutated receptor (Fig. 1a). Site-directed variants of a His_6_-tagged *A. thaliana* PEX5 PTS1-binding domain (residues 340–728, *At*PEX5C)^16^, selected by analysis of known crystal structures, were expressed in *Escherichia coli*. The purified proteins were incubated with a library of pentapeptides, representing known PTS1 and putative PTS1* sequences. After capture of the receptor-peptide complexes on cobalt affinity resin, the bound peptides were eluted and identified by liquid chromatography-mass spectrometry (LC-MS) analysis. Screening of single-site *At*PEX5C variants with the library of peptides was used to identify the most promising *At*PEX5C mutations and peptide combinations. These mutations were subsequently combined, thus minimizing the dimensionality of the search (Fig. 1a). This assay enabled both the rapid survey of many potential binding partners for the mutated PEX5, and the analysis of how sequence specificity of binding changed upon introduction of the mutations.

The pentapeptide library sequences were based on YQSKL, which has a nM affinity for the *At*PEX5 TPR domain^17^ and is widely used as a model PTS1 peptide^14^. This sequence was randomized at the two C-terminal positions to create a library of sequences, YQSXX, where X represents any amino acid, which was efficiently prepared using split-and-pool solid-phase peptide synthesis. A dansyl fluorophore introduced at the N-terminus of the peptide added hydrophobicity to improve retention in LC-MS analysis. Deconvolution of Leu- and Ile-containing peptides was enabled by preparation of four sub-libraries that separated these isobaric residues for characterization, although all screens were performed on the combined library. Cysteine was excluded from the library design to avoid any complications from disulfide formation, while oxidation of methionine during synthesis meant that methionine-containing peptides were also excluded from the analysis. The final library hence contained 324 sequences.

We first determined the LC retention time of each peptide present in the library using a tandem quadrupole time-of-flight mass spectrometer. By analyzing extracted ion chromatograms based upon the exact (rather than the nominal) masses of the peptide ions^18^, and using LC-MS/MS methods to distinguish positional isomers, all the peptides could be observed in the parent libraries (Supplementary Fig. 1). Unique retention times were determined for 236 of the 324 peptides; however, a lack of HPLC separation meant that the remaining peaks could only be assigned to a pair of positional isomers that co-eluted, for example dansyl-YQSKI and dansyl-YQSIK. While the difference in ionization efficiency between the peptides (up to ~ 250-fold between the weakest and the strongest signals) and the presence of overlapping peaks made the method only semi-quantitative, the high sensitivity and ease of the screen enabled rapid comparison of the sequence specificity of variant receptor proteins for the library of peptides.

**Fig. 2:**
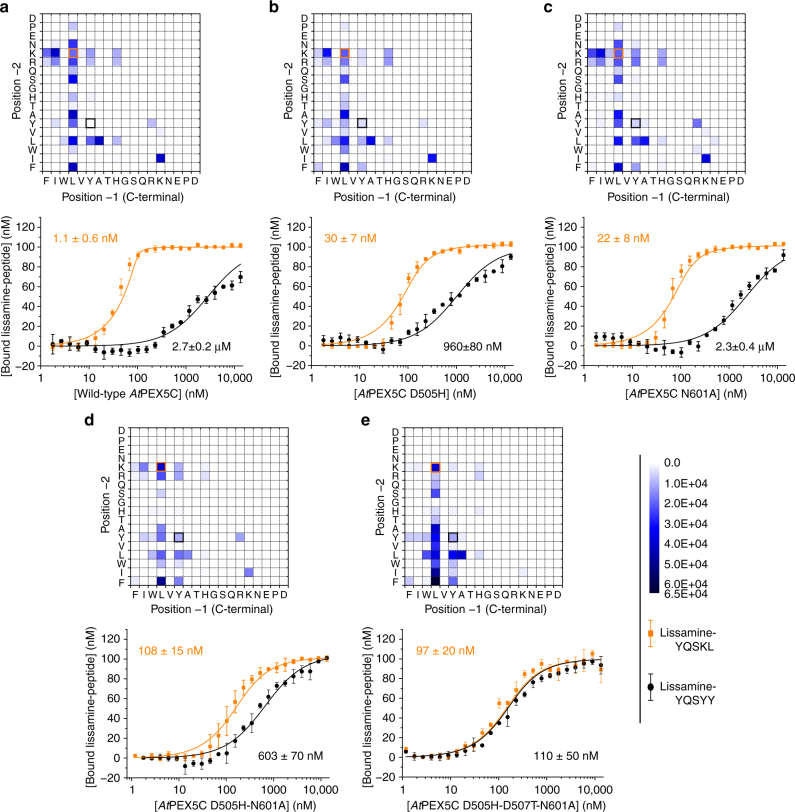
Peptide-binding selectivities for wild-type and key *At*PEX5C variants. Heat maps (showing area-under-extracted ion chromatograms (EICs) for pulled-down peptides are shown, along with fluorescence anisotropy results using lissamine-YQSKL (*orange*) and lissamine-YQSYY (*black*). Darker blue indicates increased area-under-EIC in the LC-MS analysis. **a** Wild-type *At*PEX5C. **b** *At*PEX5C D505H. **c** *At*PEX5C N601A. **d** *At*PEX5C D505H-N601A. **e** *At*PEX5C D505H-D507T-N601A. Across the series **a**–**e**, an increased affinity of protein variants to lissamine-YQSYY is observed, along with a decrease in affinity for lissamine-YQSKL. The curves represent non-linear least squares fitting to a 1:1 binding model, fitted using OriginPro. *Error bars* represent standard deviations of triplicate repeats

As anticipated, using the wild-type *At*PEX5C receptor domain, the peptides pulled down from the library revealed a strong preference for leucine at the C-terminal (-1) position (Fig. 2a): a characteristic of many native PTS1 sequences^19, 20^. A wider variety of residues were observed at the -2 position, with peptides bearing H, N, S, Q, P, W, T, A, Y, L, and F all being recovered along with the more prototypical K and R. Bioinformatic tools enable the prediction of the peroxisomal targeting efficiency of sequences (PredPlantPTS1^21, 22^), and peptides within the tested library were ranked based on their peroxisomal targeting prediction score. More than 75% of the PTS1-predicted peptides with a PredPlantPTS1 score above 0.6 (indicating moderate peroxisomal import) were pulled down by the wild-type *At*PEX5C receptor.

### Selection of PEX5* variants

Inspection of a model of the *At*PEX5C:PTS1 interaction generated from known crystal structures^14, 23–26^ identified residues close to the PTS1-binding site as targets for mutagenesis. Thirty-four receptor variantswere screened (Supplementary Table 1), and the patterns of peptides that were pulled down from the library of 324 peptides were visualized as heat maps (Fig. 2 and Supplementary Figs. 2 and 3). Of particular interest were the mutations D505H and N601A, resulting in *At*PEX5C variants that bound to the sequences dansyl-YQSYY and dansyl-YQSFY (Fig. 2b, c). These sequences were not pulled down by wild-type *At*PEX5C (Fig. 2a).

To validate and quantify the effect of these mutations, a fluorescence anisotropy (FA) assay^17, 27^ was employed to determine the binding affinity of lissamine-labelled YQSKL and YQSYY to the wild-type *At*PEX5C receptor and its variants (lissamine-labelled YQSFY had poor solubility and so investigations of this sequence were not pursued). The affinity of the *At*PEX5C receptor for lissamine-YQSKL, determined using the FA assay, was 1.1 ± 0.6 nM (errors represent the standard deviation of triplicate repeats), while lissamine-YQSYY had only a very weak affinity (2.7 ± 0.2 μM) (Fig. 2a). By contrast, the D505H variant of *At*PEX5C had an improved affinity of 960 ± 80 nM for lissamine-YQSYY, while the affinity for lissamine-YQSKL was reduced to 30 ± 7 nM (Fig. 2b). The N601A variant bound lissamine-YQSYY similarly to wild-type *At*PEX5C (2.3 ± 0.4 μM), while the affinity for lissamine-YQSKL was 22 ± 8 nM (Fig. 2c). Combination of these mutations, to generate D505H-N601A, resulted in an even greater change: the affinity for lissamine-YQSYY increased to 603 ± 70 nM, while the affinity for lissamine-YQSKL decreased to 108 ± 15 nM (Fig. 2d).

As residue D505 is close to the lysine side chain of YQSKL, changing this aspartate residue to histidine should remove this electrostatic interaction and create a pocket more suitable for an aromatic residue. We predicted that further decrease in the negative charge in this area may improve selectivity. Therefore, a D→T mutation was introduced at a nearby position 507, creating the variant D505H-D507T-N601A (*At*PEX5C*). This enhanced the affinity and selectivity for the variant peptide sequence to the extent that lissamine-YQSYY bound with an affinity almost identical to lissamine-YQSKL (110 ± 50 nM vs. 97 ± 20 nM) (Fig. 2e). Bioinformatic tools^21, 22^ typically also include the upstream context when predicting the in vivo peroxisomal import competence of a sequence. We hence anticipated that a further improvement in the efficacy of YQSYY would be achievable in vivo by placing it downstream of appropriate residues, to create a high-affinity PTS1*^28^.

### An optimized PEX5*-PTS1* pair in vivo

**Fig. 3:**
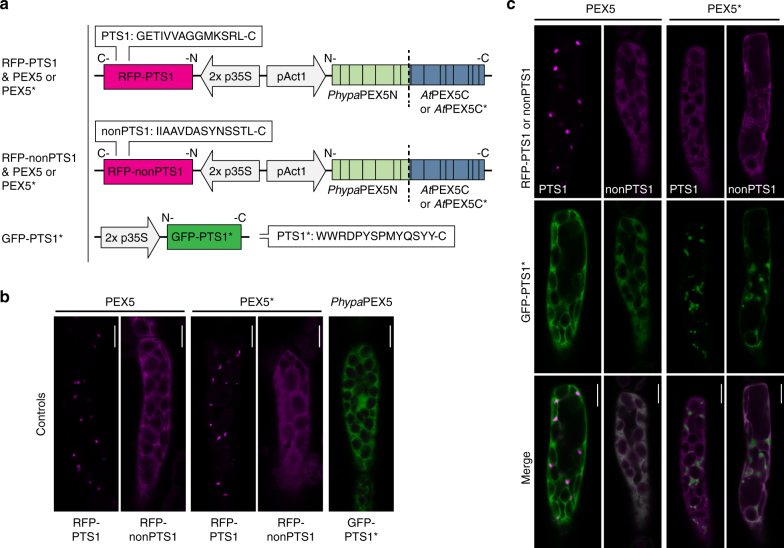
In vivo transient expression. **a** Constructs produced for the in vivo expression in *P. patens*. RFP-PTS1 or nonPTS1 was on the same vector as the hybrid receptor (*Phypa*PEX5N–*At*PEX5C (PEX5 or PEX5*)), and GFP-PTS1* was on a separate vector. **b**, **c** 36–48 h transient expression of constructs in *P. patens* cells. **b** Control experiments, expressing either RFP-PTS1 or -nonPTS1 & receptor alone, or GFP-PTS1* alone. GFP and RFP channels (merged) are shown for these images. **c** Co-transformation of both vectors (shown in part **a** of the figure). PEX5* imports GFP-PTS1* into peroxisomes, whereas PEX5 does not (under any of the conditions tested). *Scale bars*, 10 µm

With new receptor-targeting signal selectivities developed, the functionality of the mutated receptor was tested in vivo in *Physcomitrella patens*. This moss is an excellent model organism as it grows on a simple medium, has a fully sequenced genome that can be easily engineered through homologous recombination, is highly amenable to microscopic analysis, and is established as a bioreactor in biotechnological applications^29–31^. The TPR domain sequences of both the wild-type *At*PEX5 receptor and the *At*PEX5* receptor (D505H-D507T-N601A) were combined with the N-terminus of the wild-type *Phypa*PEX5 sequence to create the two hybrid receptors PEX5 and PEX5*. This approach ensured that receptor docking and recycling functions, which are a property of the PEX5N-terminal domain, were not compromised as a result of any potential cross-species incompatibility. Fluorescent reporter proteins RFP and GFP were used for in vivo testing of the PEX5*-PTS1* pair. While strong PTS1 tripeptides are both necessary and sufficient for peroxisome targeting, bioinformatics, mutational, and structural analysis all point to enhancing roles for the immediately upstream amino acids^11–13^. Therefore, RFP was modified to append the following: (1) the C-terminal 14 amino acids from a *P. patens* predicted peroxisomal short-chain dehydrogenase/reductase (Pp3c18_20320) (GETIVVAGGMKSRL; ‘PTS1’), or (2) the 14 C-terminal amino acids from an inositol phosphatase (Pp3c3_21240), which is predicted to be non-peroxisomal (IIAAVDASYNSSTL; ‘nonPTS1’) to serve as a negative control. GFP was modified by appending the sequence YQSYY including enhancing upstream residues (WWRDPYSPMYQSYY), to form an equivalent length and predicted higher affinity PTS1* at its C-terminus. Targeting predictions used the PredPlantPTS1 server^21, 22^. The genes encoding the hybrid receptors PEX5 or PEX5* were present on the same vector as the RFP reporter, which ensured that all cells with RFP signal also expressed the untagged receptor (Fig. 3a). This was essential, as tagging PEX5 with a bulky protein at its C-terminus blocks re-export^32^ and a large N-terminal tag could potentially interfere with docking or ubiquitination.

When plasmids containing the RFP-PTS1 sequence were transiently expressed in *P. patens* cells, distinct punctate bodies were visible using fluorescence microscopy. This indicates import of RFP-PTS1 into the peroxisome (Fig. 3b). Import was functional regardless of whether the plasmid contained the PEX5 or PEX5* receptor (Fig. 3b, Supplementary Figs. 4b, 5b). The efficient import observed in the presence of PEX5* can be accounted for by the endogenous *Phypa*PEX5, as bombardment of moss with the RFP-PTS1 gene in the absence of any additional receptor on the plasmid also results in similar peroxisomal fluorescence (Supplementary Fig. 6a). Conversely, bombardment of *P. patens* cells with GFP-PTS1* resulted in cytosolic localization of the green fluorescence, indicating that GFP-PTS1* is not recognized by the endogenous *Phypa*PEX5 (Fig. 3b, Supplementary Fig. 6b).When RFP was appended with the nonPTS1 sequence, the red fluorescence was observed uniquely in the cytosol, regardless of whether PEX5 or PEX5* was present on the plasmid, showing that PEX5* did not recognize this nonPTS1 sequence.

The ‘RFP & receptor’-containing plasmid was then mixed with GFP plasmid prior to bombardment into *P. patens* cells. When the plasmid containing RFP-PTS1 and PEX5 was co-bombarded with GFP-PTS1*, red fluorescence was clearly localized in punctate peroxisomes in most cells (Supplementary Fig. 7b); however, the green fluorescence was almost completely cytosolic (Fig. 3c). In contrast, in the presence of the PEX5* receptor, distinct peroxisomal localization of GFP-PTS1* was clearly observed, while the fluorescence from RFP-PTS1 was mostly cytosolic (Supplementary Fig. 8b), demonstrating a switch in import selectivity. RFP-nonPTS1 remained cytosolic in all experiments.

**Fig. 4:**
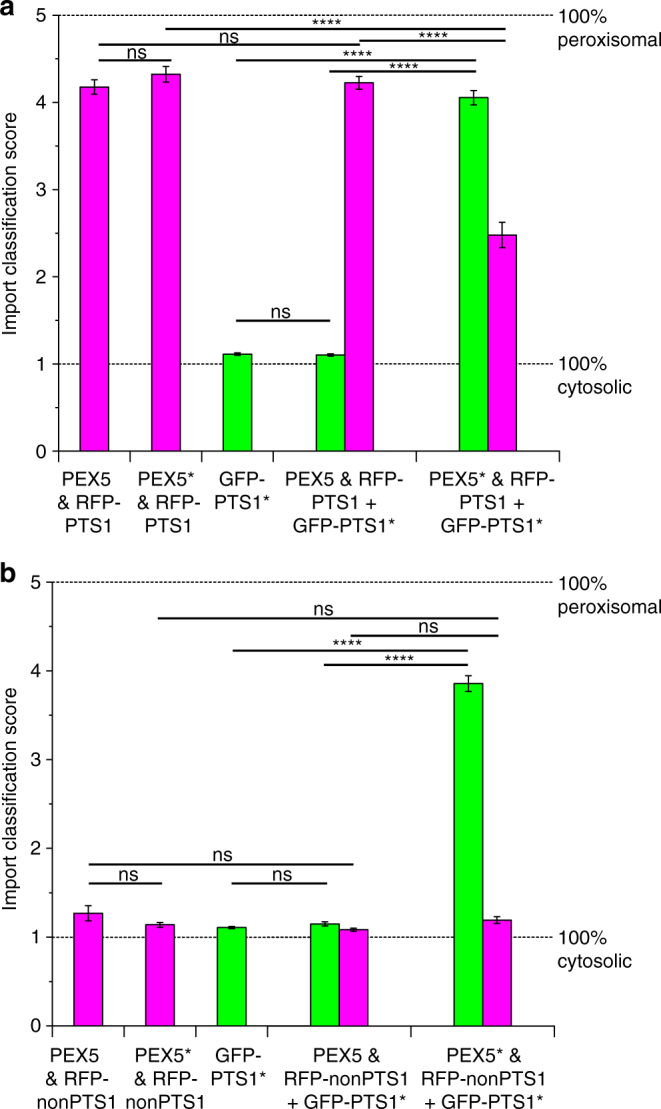
Quantitative analysis of peroxisome protein import. **a**, **b** Transient expression of constructs in *P. patens* cells. **a** RFP-PTS1 & PEX5, RFP-PTS1 & PEX5*, **b** RFP-nonPTS1 & PEX5, or RFP-nonPTS1 & PEX5*, all with and without GFP-PTS1*. Significant peroxisomal import of GFP-PTS1* was observed when PEX5* was expressed in *P. patens*, regardless of which RFP reporter was present. No such increase was seen in the absence of PEX5*. There was also a significant decrease in RFP-PTS1 import upon PEX5* expression. *Error bars* represent standard errors, *n* = 84–115 (Supplementary Table 2). ns not significant; ****P < 0.0001, determined by a Kolmogorov–Smirnov test on the original histograms (Supplementary Fig. 9, Supplementary Tables 3, 4)

To provide a quantitative analysis, the intracellular distribution of fluorescent markers was ranked on a scale of 1–5, where 5 represented complete peroxisomal localization and 1 represented fully cytosolic localization. Each image was independently classified by at least 7 assessors and monochrome images were assessed blind without the knowledge of which plasmid combination had been used, or whether red or green channels were being viewed. More than 80 images were used to generate the final classification averages for each plasmid combination (Fig. 4, Supplementary Table 2). Histograms of the spread of classifications of images were also generated to compare the distribution of outcomes (Supplementary Fig. 9).

This larger analysis reinforces the qualitative observations of individual images. Experiments in the absence of GFP-PTS1* showed that RFP-PTS1 is imported into peroxisomes (score > 4) regardless of the presence of the additional *PEX5* or PEX5* gene (Fig. 4a). Similarly, expressing GFP-PTS1* alone results in no peroxisomal import (score ~ 1) in the absence of the PEX5* gene. When both plasmids are introduced, there is a clear switch in the import selectivity of cells transiently expressing the PEX5* receptor when compared to those transiently expressing the PEX5 receptor (Fig. 4a). Co-expression of PEX5 with RFP-PTS1 and GFP-PTS1* recapitulates the phenotypes observed when the plasmids were introduced individually (RFP-PTS1 localization 4.23 ± 0.07, GFP-PTS1* localization 1.10 ± 0.01; errors represent the standard error across the images analyzed — see Supplementary Table 2 for image numbers). However, upon expression of PEX5*, cells are newly able to efficiently direct GFP-PTS1* to peroxisomes (GFP-PTS1* localization 4.05 ± 0.08). Interestingly, import of RFP-PTS1 was strongly reduced, though not completely cytosolic (localization 2.48 ± 0.15), but this was dependent on the co-expression of GFP-PTS1*, as PEX5* expression by itself did not affect RFP-PTS1 import (localization 4.32 ± 0.09) (Fig. 4a). These data suggest that GFP-PTS1*-loaded PEX5* outcompeted the endogenous receptor-cargo complexes for some limiting step in the import pathway. Strong import of GFP-PTS1* by PEX5* was also observed when the non-competitive cargo RFP-nonPTS1 was co-expressed (localization 3.86 ± 0.09) (Fig. 4b).

Confirmation that GFP-PTS1* was indeed delivered to the peroxisome (rather than an alternative compartment) by PEX5* was obtained by careful analysis of images in which partial import of RFP-PTS1 is also seen. Clear co-localization of red and green fluorescence is observed in the punctate structures, which is strongly indicative of a peroxisomal localization (Supplementary Figs. 8b, 10d). We recently reported that genetic knockout of *PEX11*, a gene involved in peroxisome division, in *P. patens* leads to the formation of giant peroxisomes^33^. Additional evidence for peroxisomal localization of the fluorophores was obtained using this mutant line. Bombardment of the *pex11ko* line with the plasmids containing GFP-PTS1*, RFP-PTS1 and PEX5 led to the localization of red fluorescence in large peroxisomes, while the GFP remained cytosolic. When the fluorescent reporters were expressed in the presence of PEX5*, the selectivity was switched so that the large peroxisomes selectively imported the GFP, with some cases of co-localization with RFP (Supplementary Fig. 10a–c).

## Discussion

Manipulation of the substrate specificity of enzymes, often by directed evolution, is well established for both catalysis and the understanding of metabolic pathways (such as kinase engineering to accept ATP analogues)^34–37^. However, the modification of protein–protein interactions is far less reported^38–41^. Here we have demonstrated the engineering of a protein–protein interaction that can support functional peroxisomal protein trafficking in vivo. Native peroxisomal protein import is characterized by the high plasticity of the PEX5 receptor, which can accommodate a number of different natural PTS1 sequences. This sequence tolerance was a complicating factor in the search for a novel targeting signal-receptor pair. We built on previous work showing good correlation between the strength of binding of signal peptides and import efficiency^17^ to demonstrate that an approach of selecting mutations and screening libraries of peptides using LC-MS can quickly identify those sequences with the greatest potential to act as orthogonal motifs. Iterative development led to the variant *At*PEX5C*, which showed high affinity in vitro for the pentapeptide YQSYY (which bound only weakly to wild-type *At*PEX5C). *At*PEX5C* also showed a significantly decreased affinity for a prototypical PTS1 pentapeptide. Bioinformatic tools guided further optimization of upstream residues, enhancing the predicted ability of the orthogonal targeting signal (PTS1*) to promote cargo import in vivo in moss cells. Transient expression of fluorescent reporter proteins tagged with PTS1 or PTS1* sequences and their cognate receptors enabled the intracellular distribution of cargo proteins to be visualized.

The high affinity of PEX5* for the PTS1* sequence resulted in preferential import of PTS1*-tagged proteins at the expense of native PTS1 sequences, even when the cell was still expressing endogenous *Phypa*PEX5, with the PEX5*-PTS1* pairing outcompeting the endogenous pathway. Interestingly, this competition only occurred in the presence of the PTS1* cargo, suggesting that there is selection for cargo-loaded receptor at the peroxisomal membrane. PEX5 insertion into purified rat liver peroxisomes was shown to be cargo-dependent by Gouveia and colleagues^42^, although recombinant cargo-free PEX5 can bind its purified docking partner PEX14 in vitro^16^. It will be interesting to explore the mechanistic basis of this selectivity.

The ability of the PEX5*-PTS1* pair to outcompete import of endogenous PTS1 proteins provides a convenient tool to manipulate peroxisomal protein import in vivo, thereby allowing measurement of rates of import and half-life of cytosolic and peroxisomal proteins — parameters which are useful for modelling in vivo peroxisome protein trafficking, and in systems biology. Our results also suggest that this mutated receptor and targeting signal pairing can be used to drive a switch in peroxisomal function, allowing import of user-specified proteins without competition from endogenous proteins. This switch in peroxisomal function could bring about a repurposing of the peroxisome within the cell for use as a novel intracellular compartment. While creating a true designer organelle that exists in parallel with naturally occurring compartments (including the peroxisome) will require manipulation of several other aspects of organelle biogenesis, this work demonstrates the creation of a new and orthogonal trafficking pathway, an essential component for the realization of this goal in synthetic biology.

## Methods

DNA and protein sequences of key constructs used in this study are shown in Supplementary Figs. 11–24.

### Site-directed mutagenesis of *At*PEX5C

The gene encoding His_6_-*At*PEX5 (340–728), referred to as *At*PEX5C, contained within the pET-28b vector, is described by Lanyon-Hogg and colleagues^16^. Mutations were introduced using a QuikChange Lightning site-directed mutagenesis kit (Agilent Technologies) according to manufacturer’s instructions. Transformation of each *Dpn*I-treated mutagenesis reaction into XL10-Gold cells was performed, followed by DNA purification and confirmation of mutagenesis by sequencing (Beckman Genomics).

### Expression and purification of *At*PEX5C and variants

The *At*PEX5C DNA (wild-type or mutant) was transformed into *E. coli* BL21-Gold (DE3) cells for efficient protein expression. Overnight growth of single colonies in 5 ml selection medium (Lysogeny broth containing kanamycin at 50 mg l^−1^) at 37 °C was performed, followed by inoculation of 20 µl of this culture into 1 ml selection media at 37 °C, and growth for 8 h (‘day culture’). Autoinduction medium (tryptone (1% w/v), yeast extract (0.5% w/v), NaOH (1 mM), (NH_4_)_2_SO_4_ (25 mM), KH_2_PO_4_ (50 mM), Na_2_HPO_4_ (50 mM), MgSO_4_ (1 mM), glycerol (0.5% w/v), glucose (0.05% w/v), α-lactose (0.2% w/v), kanamycin (100 mg l^−1^)) was inoculated with day culture (250 µl into 500 ml of autoinduction medium) and incubated at 28 °C for 18 h. Cells were collected by centrifugation and resuspended in wash buffer (Na_2_HPO_4_ (50 mM), NaCl (300 mM), 2-mercaptoethanol (10 mM), glycerol (15% v/v), pH 8.0 with 1 M NaOH), 40 ml per 500 ml original autoinduction culture). Protein purification was performed following cell lysis by two passes at 30 kpsi through a cell disruptor (Constant Systems, TS Series Benchtop Cell Disruptor). The supernatant of the lysed cells was incubated with Co-NTA resin (Thermo Scientific, 500 μl settled resin per 40 ml supernatant) at 4 °C, for 1 h, with agitation. The supernatant was removed, the resin was washed with wash buffer (4 × 15 ml washes) and the protein eluted from the Co-NTA resin with elution buffer (wash buffer containing 100 mM imidazole, 2 ml per 500 ml original autoinduction culture). The resulting protein identity and purity was confirmed using SDS-PAGE and ESI-LC-MS (Supplementary Fig. 25).

### Lissamine-labelled peptide synthesis

Lissamine sulfonyl chloride-labelled peptides were prepared using Fmoc solid-phase peptide synthesis: 100 mg of leucine- or tyrosine-loaded 2-chlorotrityl chloride resin (Merck) was added to a fritted reaction vessel. The resin was swelled in dimethylformamide (DMF; 5 ml) for 1 h, and then the reaction vessel was drained. DMF washes were performed (3 × 2 ml × 2 min), followed by washes with 20% piperidine in DMF (5 × 2 ml × 2 min), and DMF (5 × 2 ml × 2 min). FmocBoc Lys or Fmoc^t^Bu Tyr (5 e.q.) in DMF (1 ml) was added, along with *O*-(1*H*-6-chlorobenzotriazole-1-yl)-1,1,3,3-tetramethyluronium hexafluorophosphate (HCTU; 5 e.q.) in DMF (1 ml) and diisopropylethylamine (DIPEA; 10 e.q.). The reaction was agitated for 1 h at RT then washed with DMF, deprotected (20% piperidine in DMF) and washed (DMF). The remaining couplings, of the S, Q, and Y residues, were performed using similar procedures. The N-terminal position was capped by coupling with lissamine rhodamine B sulfonyl chloride (Thermo Fisher Molecular Probes). Lissamine (3 e.q.) was added to the resin-bound peptide (1 e.q.) at 0 °C, and anhydrous DIPEA (10 e.q.) was added before stirring the solution overnight in the absence of light. The solution was filtered from the resin, which was then washed with DMF (3 × 2 ml), dichloromethane (DCM) (3 × 2 ml), and MeOH (3 × 2 ml) and dried under reduced pressure. The peptides were cleaved from the solid resin support using a cleavage cocktail of trifluoroacetic acid (TFA), H_2_O, and triisopropylsilane (95:2.5:2.5), which was added to dried resin (500 µl per 25 mg resin) and agitated for 1 h at RT. The cleavage mixture was filtered drop-wise into cold diethyl ether (1:100) and precipitated peptide was collected by centrifugation. Three diethyl ether washes were performed. The combined diethyl ether layers were extracted with water and the aqueous layer combined with the precipitated peptide, lyophilized and purified by reverse phase HPLC.

### Peptide library synthesis

The peptide libraries were prepared using Fmoc solid-phase peptide synthesis using a split and pool approach. Side-chain protecting groups were (S,T,Y):^t^Bu, (D,E):O^t^Bu, (H,N,Q):Trt, (W,K):Boc, R:Pbf. Four sub-libraries were prepared to enable deconvolution of isobaric peptides. The sub-libraries were combined for the pull-down assays. The four sub-libraries had the following sequences:

Sub-library 1: dansyl-[Y]-[Q]-[S]-[D/F/G/H/I/K/N/P/S/V]-[D/F/G/H/I/K/N/P/S/V]-CO_2_H

Sub-library 2: dansyl-[Y]-[Q]-[S]-[D/F/G/H/I/K/N/P/S/V]-[A/E/L/M/Q/R/T/W/Y]-CO_2_H

Sub-library 3: dansyl-[Y]-[Q]-[S]-[A/E/L/M/Q/R/T/W/Y]-[D/F/G/H/I/K/N/P/S/V]-CO_2_H

Sub-library 4: dansyl-[Y]-[Q]-[S]-[A/E/L/M/Q/R/T/W/Y]-[A/E/L/M/Q/R/T/W/Y]-CO_2_H

Peptide sub-libraries were synthesized by first adding 30 mg of each of the required preloaded 2-chlorotrityl chloride resin (Merck) bearing the required -1 residues into a fritted reaction vessel. The pooled resin was swelled in DMF (5 ml) for 1 h, and then the reaction vessel was drained. DMF washes were performed (3 × 2 ml × 2 min), followed by washes with 20% piperidine in DMF (5 × 2 ml × 2 min), and DMF (5 × 2 ml × 2 min). Resin was washed with DCM (3 × 2 ml × 2 min) and dried in vacuo before mixing thoroughly and splitting equally between reaction vessels for coupling of each of the amino acids at position -2. To each aliquot of resin, a different Fmoc-protected amino acid (5 e.q.) in DMF (1 ml) was added along with HCTU (5 e.q.) in DMF (1 ml) and DIPEA (10 e.q.). The reaction was agitated for 1 h at RT. Following washing with DMF, all aliquots of resin were pooled for the deprotection (20% piperidine in DMF) and wash (DMF) steps. The remaining couplings, of the S, Q, and Y residues, were performed on the pooled resin using similar procedures. The N-terminal position was capped by coupling with dansyl chloride (Sigma-Alrich). The resin-bound peptide library (1 e.q.) was swelled in DMF (2 ml) for 1 h. DMF was drained from the reaction vessel. DIPEA (6 e.q.) was added to dansyl chloride (5 e.q.) in DMF (2 ml), and the solution was well mixed, incubated at RT for 10 min, then added to the resin-bound peptide library (1 e.q.) and stirred overnight. The solution was filtered from the resin, which was then washed with DMF (3 × 2 ml), DCM (3 × 2 ml) and MeOH (3 × 2 ml), and dried under reduced pressure. The peptides were cleaved from the solid resin support using a cleavage cocktail of TFA, H_2_O, and triisopropylsilane (95:2.5:2.5), which was added to dried resin (500 µl per 25 mg resin) and agitated for 1 h at RT. The cleavage mixture was filtered drop-wise into cold diethyl ether (1:100) and precipitated peptide was collected by centrifugation. Three diethyl ether washes were performed. The combined diethyl ether layers were extracted with water and the aqueous layer combined with the precipitated peptide, lyophilized and re-suspended in sterile water to the desired concentration.

### Pull down of binding peptides by *At*PEX5C protein

Purified protein (final concentration: 12.5 µM) was added to the combined peptide library (final concentration: 500 nM each peptide in the library (concentration calculated based on the average molecular weight of the peptides in each sub-library)) in a 500 µl reaction mixture in wash buffer (Na_2_HPO_4_ (50 mM), NaCl (300 mM), 2-mercaptoethanol (10 mM), glycerol (15% v/v), pH 8.0 with 1 M NaOH), and incubated at 4 °C for 1 h with agitation. This protein-peptide mixture was added to Co-NTA resin (100 µl settled resin per 500 µl reaction) and this was incubated at 4 °C for 1 h with agitation. Supernatant was removed and 500 µl wash buffer was added to the resin (4 × 500 µl washes), followed by wash buffer containing 5 mM imidazole (3 × 500 µl washes). Remaining bound peptides were effectively eluted from the protein by incubating the resin with wash buffer containing 6 M urea (300 µl) at 4 °C for 30 min with agitation, to unfold the protein. Eluate was collected in tapered vials and 10 µl was injected for analysis by ESI-LC-MS.

### LC-MS analysis of peptides

Electrospray ionization LC-MS was performed using a Bruker MaXis Impact time-of-flight mass spectrometer in the positive ion mode. Analytical column used: Waters Acquity UPLC Peptide CSH C18 column 130 Å, 1.7 μm stationary phase (column dimensions 2.1 × 100 mm) fitted with the corresponding Vanguard pre-column guard (guard column dimensions 2.1 × 5 mm). UPLC was performed using a Dionex Ultimate 3000 HPLC, with the following solvents: (A) water + 0.1% formic acid; (B) acetonitrile + 0.1% formic acid. Gradient timetable (flowrate 0.7 ml min^−1^, linear gradients between points): −1.3 min = 99:1 A:B (pre-equilibration); 0 min = inject; 0.3 min = 99:1 A:B; 1.5 min = 80:20 A:B; 2.5 min = 78:22 A:B; 4.5 min = 70:30 A:B; 5.5 min = 60:40 A:B; 6 min = 5:95 A:B; 7.5 min = 1:99 A:B. Calibration of the mass spectrometry instrument was performed using sodium formate, injected at the end of each run. The following analysis steps were automated using the VBScript functions within the Bruker DataAnalysis software, or using custom VBA routines in Microsoft Excel.

### Generation of extracted ion chromatograms

Following acquisition of the data, an extracted ion chromatogram was generated for ions corresponding to each of the unique formulas contained within the peptide library. (M + H)^+^ ions were considered for all peptides, and (M + 2H)^2+^ peptides were also considered for peptides containing R and K residues. The extracted ion chromatograms were created with a width of ± 0.008 Da. The chromatograms were smoothed and integrated, and the retention times and areas were used in subsequent analysis.

### Generation of reference retention times for peptides

Each peptide sub-library was analyzed using the LC-MS method listed above and an identical chromatographic run in which the mass spectrometer provided automated MS/MS data, using collisional induced fragmentation in the quadrupole. Based on the original composition of the library, the accurate mass of each of the peptides (represented by the appearance of a peak in the extracted ion chromatograms) and the observation of the y_1_ fragment ions, unique retention times could be determined for the vast majority of peptides within the mixture. Where no chromatographic separation was observed, the retention time was associated with both sequences. The full reference data set is available in Supplementary Data 1 and 2.

### Identification of peptides from pull-down experiments

The extracted ion chromatogram data were compared with the reference mass-retention time list to identify the best match between the data observed and the reference set. A small retention time drift of up to ± 5 s was allowed in this matching process, and the best match was determined by whichever offset gave the lowest minimal RMS deviation of retention times between sets. Typically retention time offsets were≤ 1 s. Once identified, the EIC areas were plotted against the peptide sequences in heat map plots using OriginPro.

### Fluorescence anisotropy

FA assays used an Envision^TM^ 2103 multilabel plate reader (Perkin Elmer) and were performed in 384-well microtiter plates (Black Perkin Elmer Optiplates) as follows. Four solutions were prepared (A: FA buffer (HEPES (20 mM), NaCl (150 mM), pH 7.5); B: Blocking solution: FA buffer containing 0.32 mg ml^−1^ of porcine gelatine; C: 40 µM solution of *At*PEX5C or variant in FA buffer; D: Fluorescent tracer solution: 200 nM solution of lissamine-YQSKL or lissamine-YQSYY in FA buffer). A total of 80 µl of solution B was added to all wells and the plate sealed and incubated at 4 °C overnight. A volume of 60 µl of solution B was removed from each well and 40 µl of solution C was added to wells in column 1 of the plate, followed by agitation and transfer of 40 µl to the corresponding wells in column 2. This process was repeated up to column 23 (40 µl was discarded from column 23 wells after agitation, leaving column 24 with no protein solution). Finally, solution D (20 µl per well) was then added to 3 rows and FA buffer added to the other three rows to act as blanks. The plate was incubated at 25 °C with linear shaking for 20 min and then read using a Perkin Elmer Envision Plate reader using the following optics: Excitation filter 531 nm (25 nm bandwidth), 555 nm polarized dichroic mirror; emission was detected in two separate channels each with 595 (60) nm filters but with orthogonal polarization (S and P polarizers). Thirty flashes were used per measurement. The data were blank-corrected and processed to give a blank-corrected anisotropy using Eq. (1).

1 $$r = 1000 \times \frac{{s - gp}}{{s + g2p}}$$

where *s* and *p* are the blank-corrected intensities in the *s* and *p* polarised channels and the instrument response factor (*g*) was set to 1.16 on the instrument.

The anisotropy was converted to the amount of tracer bound (*L*_B_) using Eq. (2).

2 $$\frac{{{L_{\rm{B}}}}}{{{L_{\rm{T}}}}} = {\left[ {\frac{{\lambda \left( {{r_{{\rm{max}}}} - r} \right)}}{{\left( {r - {r_{{\rm{min}}}}} \right)}} + 1} \right]^{ - 1}}$$

where *L*_B_ is the concentration of fluorescent tracer bound to PEX5, *L*_T_ is the total tracer concentration (100 nM), *r*_max_ is the maximum anisotropy and *r*_min_ the minimum anisotropy observed in the titration. *λ* reflects the difference in quantum yields of the bound and free states, which was determined to be 1.

The *K*_d_ for the tracer was then determined by plotting the bound tracer concentration against the total protein concentration and fitting to Eq. (3) using non-linear least squares in OriginPro. The error in *K*_d_ is obtained from the fitting error within the procedure.

3 $${L_{\rm{B}}} = \frac{{\left( {{L_{\rm{T}}} + {P_{\rm{T}}} + {K_{\rm{d}}}} \right) - \sqrt {{{({L_{\rm{T}}} + {P_{\rm{T}}} + {K_{\rm{d}}})}^2} - 4{L_{\rm{T}}}{P_{\rm{T}}}} }}{2}$$

where *L*_B_ is the concentration of fluorescent tracer bound to PEX5, and *L*_T_ is the total tracer concentration. *P*_T_ is the total concentration of PEX5.

### Moss growth and transient expression

Protonemal tissue of *Physcomitrella patens* (Gransden strain) was subcultured in BCDAT growth media^43^ containing 0.6% plant agar, and 5- to 6-day-old tissue was used to perform particle bombardment for transient expression. Plasmid DNA 0.7–1 µg was coated onto tungsten M17 particles and bombarded into the moss tissue at 900 psi. Moss tissue was grown under continuous illumination (ca. 50–55 μmol m^−2^ s^−1^) at 25 °C, and imaging was conducted 36–48 h following bombardment.

### Microscopy

Images for classification were generated using fluorescence microscopy with a Zeiss AxioImager M2 microscope, containing Nomarski optics and a HXP120C light source. GFP and RFP were detected using Zeiss filters with Semrock narrow-band pass filters. Images were then captured with an Axiocam MRM camera through the ZEN2 software.

For confocal microscopy, a Zeiss LSM 800 laser-scanning inverted microscope with a 63× oil immersion objective was used. GFP and RFP were excited with a 488 nm argon laser and a 543 nm laser, respectively. Emissions for GFP/RFP were detected by a 488/543 nm dichroic mirror and 505–530 nm/560–605 nm band pass filters. Images were generated through the ZEN2011 software and processed by Adobe Photoshop CS6 or ImageJ 1.50b.

### Classification analysis

For classification, separate grayscale images of RFP and GFP channels were saved in JPEG format. Files and associated metadata (channel and experimental conditions) were uploaded to a custom webserver, which enabled the user to view an image selected at random, and classify the phenotype observed by pressing the corresponding button on the webpage. During classification, users were unaware of any metadata (i.e., which channel or experiment was being observed). Users could classify the localization of fluorescence in each image on a scale of 1–5, where a score of 1 = fully cytosolic, 2 = ~ 75% cytosolic, 3 = mixed, 4 = ~ 75% peroxisomal and 5 = fully peroxisomal. Where more than one cell was present in an image, the cell closest to the center was classified. Classifiers could also log the absence of fluorescence or the ambiguity in which cell was to be classified. The results of each classification by each user were logged in a database for subsequent analysis.

### Data processing

The classification data were processed as follows. (1) The spread of classifications for each image were collated in Microsoft Excel. (2) Any images where more than two classifiers had indicated ambiguity (in which cell was to be classified) were removed from the data set. (3) The standard deviation of the classifications for each channel was examined. (4) Any image with a standard deviation of classification >1.1 was examined — single outlier classifications from erroneous clicks during classification were removed. (5) Images with bimodal distributions were removed from the analysis completely. Following application of this processing, any image with < 5 classifications was removed from the analysis. The application of these filters resulted in loss of only 5% of the total images used within the final classifications. Following the filtering of the data, the mean classification value for each channel for each image was determined and then the average of the mean classification for each channel across all images for each experimental condition was calculated along with the standard errors which are reported in Fig. 4. Statistical significance of the differences was determined using a Kolmogorov–Smirnov test on the original histograms showing normalized distribution of peroxisomal localization scores (Supplementary Fig. 9, Supplementary Tables 3, 4). The classification details for each image are provided in Supplementary Data 3.

### Data availability

The data sets generated and/or analyzed during the current study are available in the Research Data Leeds Repository, https://doi.org/10.5518/218; any other data are available from the authors upon reasonable request.

## Electronic supplementary material

Supplementary Data 1

Supplementary Data 2

Supplementary Data 3

Supplementary Information
