# Supplementary material for: Towards designer organelles by subverting the peroxisomal import pathway
Source: Nat Commun. 2017 Sep 6;8:454. doi: 10.1038/s41467-017-00487-7 (PMC5587766; doi:10.1038/s41467-017-00487-7)
Supplement: Supplementary file 4 — Supplementary Information [file 41467_2017_487_MOESM4_ESM.pdf]

Description of Supplementary Files

Title: Supplementary Information

Description: Supplementary Figures, Supplementary Tables, and Supplementary Methods

Title: Peer Review File

Title: Supplementary Data 1

Description: Retention times for peptides using the HPLC conditions listed in the Methods section. Peptides shaded in blue have an isobaric peptide at the same retention time – the partner peptides are listed in Supplementary Data 2.

Title: Supplementary Data 2

Description: Peptides with identical exact mass and retention times to 45 peptides highlighted in blue in the retention time-sequence list (Supplementary Data 1).

Title: Supplementary Data 3

Description: Image classification data. The table shows the classification counts and averages for each image used in generating the final averages. For controls using only one plasmid, cells are greyed out when the reporter was not present.

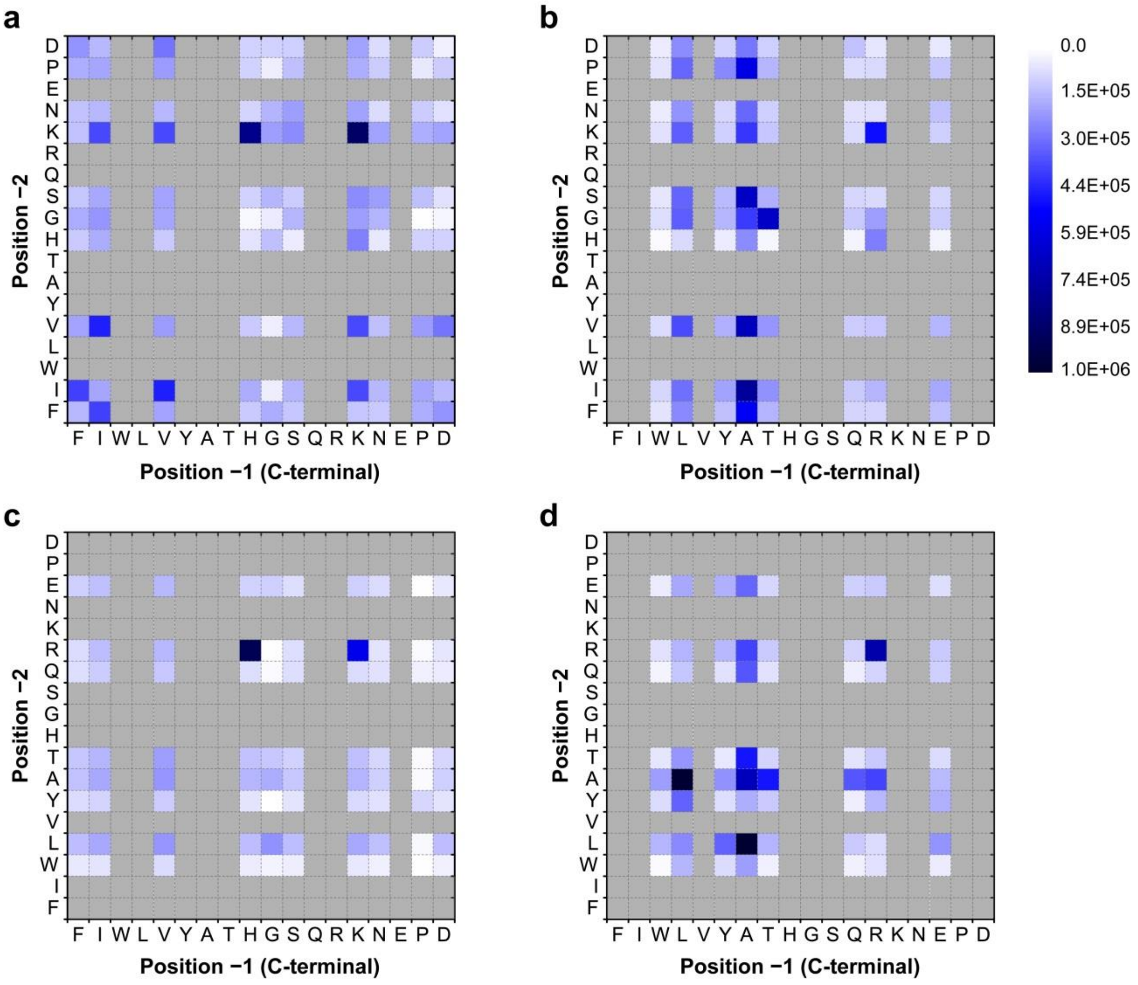

Supplementary Figure 1 | Heat maps (showing area-under-extracted ion chromatograms (EICs) for each of the four preparations of peptide sub-libraries which were combined to make the screening library. All peptides were present in each of the spectra of sub-libraries, at varying intensities. (**a**) Sub-library 1.1: Dansyl-[Y]-[Q]-[S]-[D/F/G/H/I/K/N/P/S/V]-[D/F/G/H/I/K/N/P/S/V]. (**b**) Sub-library 1.2: Dansyl-[Y]-[Q]-[S]-[D/F/G/H/I/K/N/P/S/V]-[A/E/L/Q/R/T/W/Y]. (**c**) Sub-library 2.1: Dansyl-[Y]-[Q]-[S]-[A/E/L/Q/R/T/W/Y]-[D/F/G/H/I/K/N/P/S/V]. (**d**) Sub-library 2.2: Dansyl-[Y]-[Q]-[S]-[A/E/L/Q/R/T/W/Y]-[A/E/L/Q/R/T/W/Y].

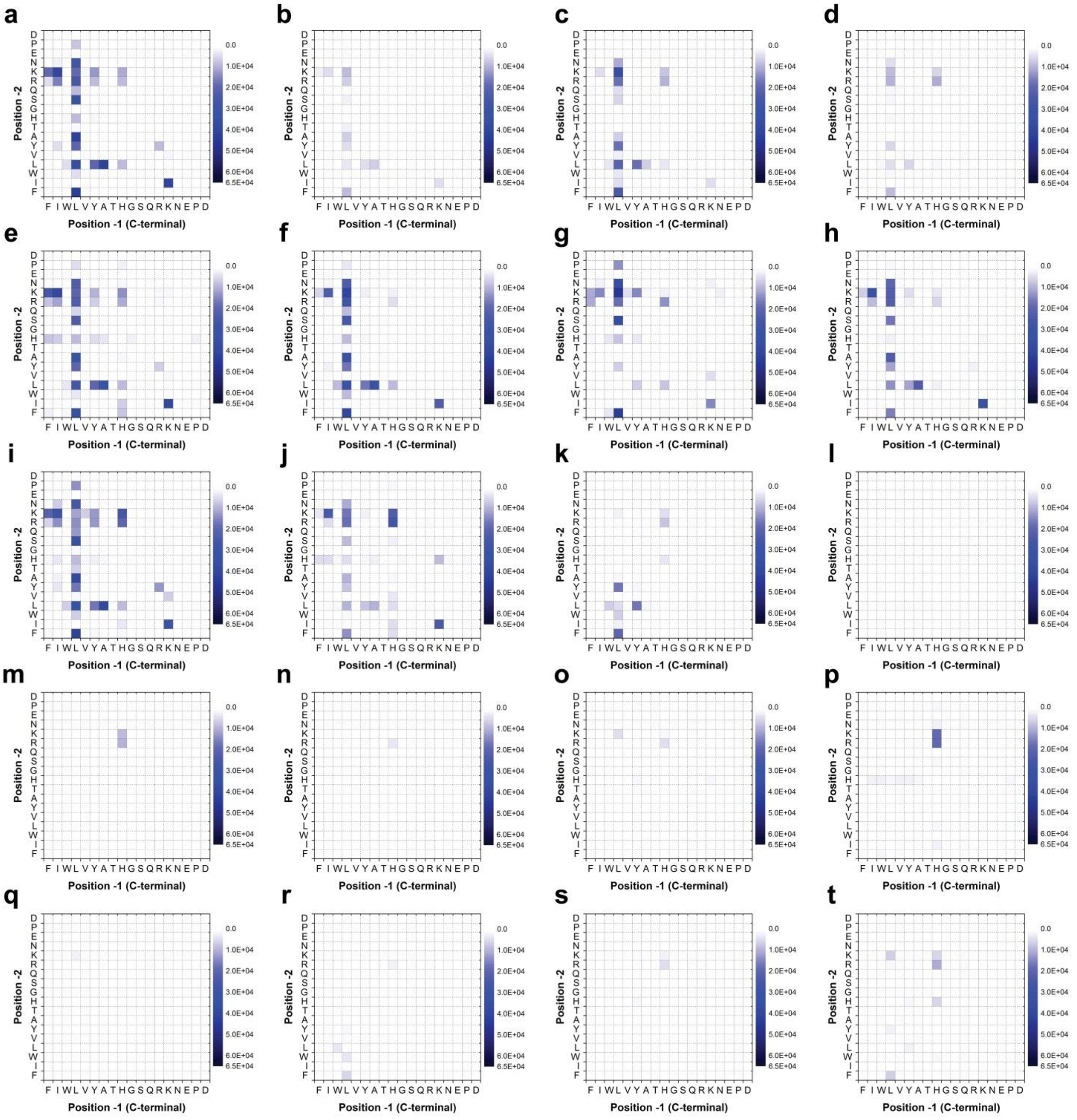

Supplementary Figure 2 | Pull-down-LC-MS heat maps for *At*PEX5C variants either with a similar peptide-binding profile to wild-type *At*PEX5C or with a background intensity of peptides pulled down. (**a**) wild-type *At*PEX5C. (**b**) D505A. (**c**) D507A. (**d**) D507K. (**e**) T536A. (**f**) E538A. (**g**) N601Q. (**h**) Y647F. (**i**) S667A. (**j**) D505H-T536W. (**k**) D505H-D507V-N601A. (**l**) D505K. (**m**) D505K-D507K. (**n**) N537A. (**o**) N628A. (**p**) R659A. (**q**) N663A. (**r**) D505F-N601A. (**s**) D505F-D507F-N601A. (**t**) D505H-N601A-N636A.

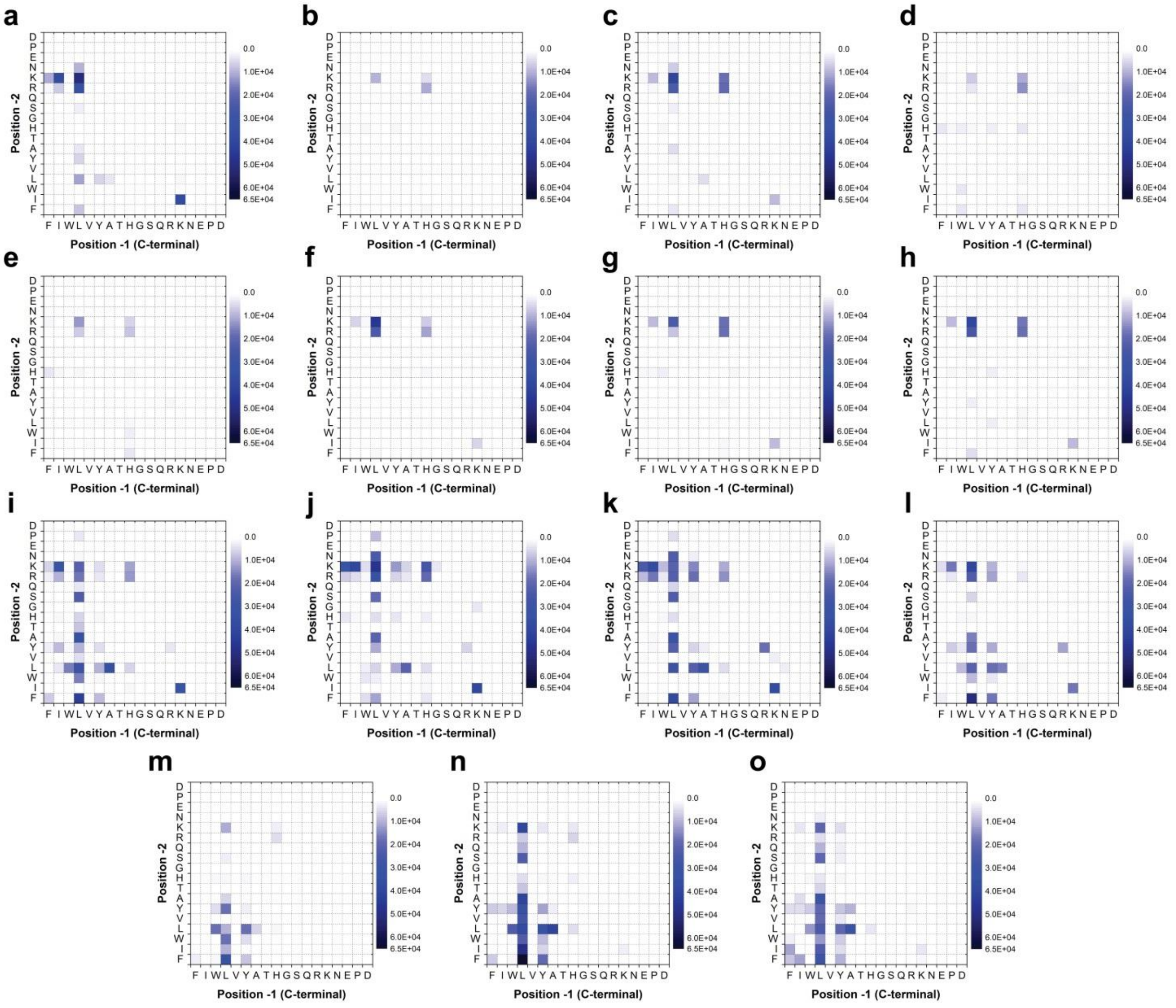

Supplementary Figure 3 | Pull-down-LC-MS heat maps for *At*PEX5C variants which either pulled down only strong PTS1 sequences or which pulled down non-PTS1 sequences.(**a**) V533A. (**b**) V533W. (**c**) T536N. (**d**) N537T. (**e**) N537Q. (**f**) F613A. (**g**) A632G. (**h**) N636A. (**i**) D505H. (**j**) T536W. (**k**) N601A. (**l**) D505H-N601A. (**m**) D505H-D507H-N601A. (**n**) D505H-D507T-N601A. (**o**) D505H-D507T-E538A-N601A.

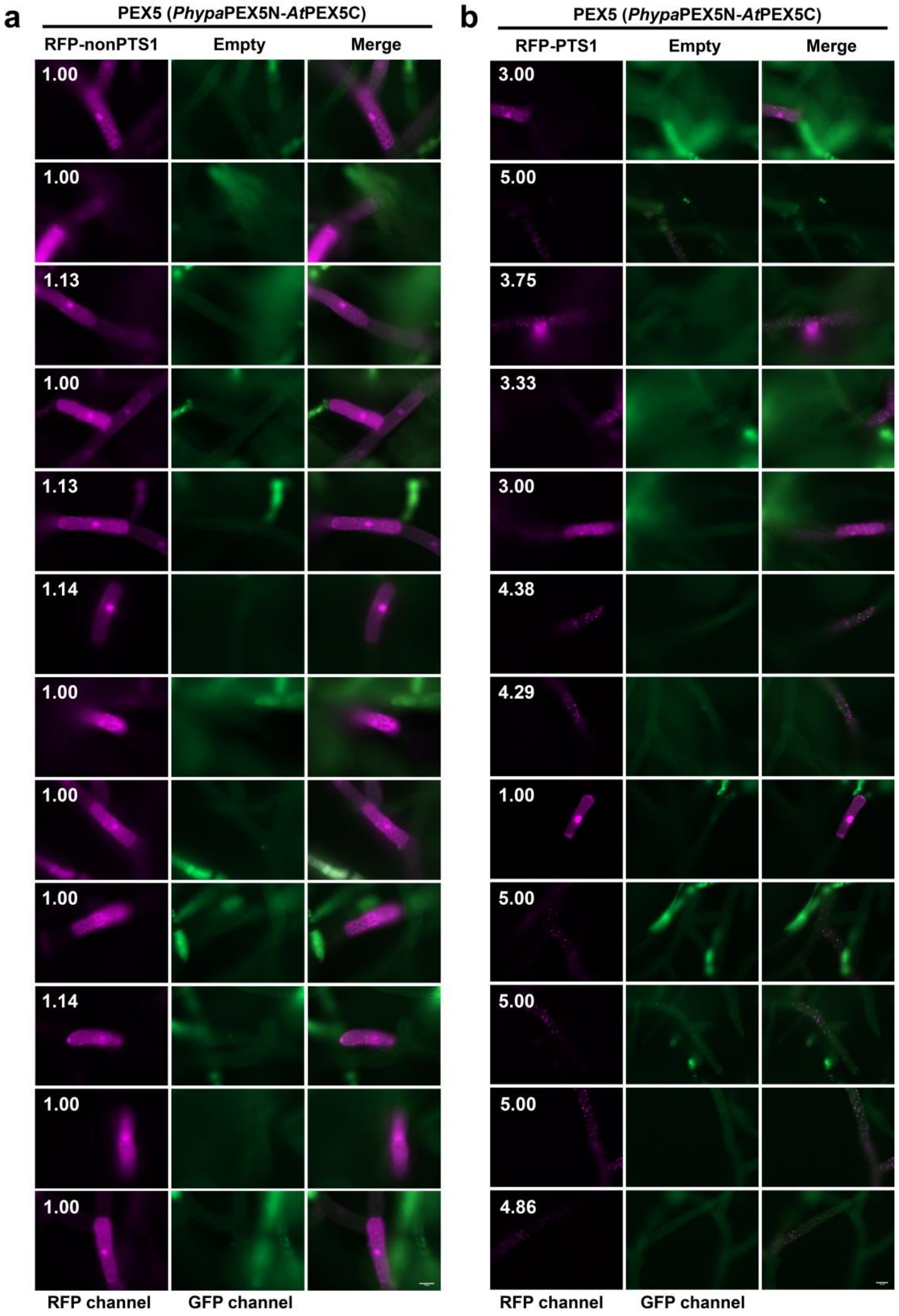

SupplementaryFigure 4 | 12 representative images of 6–7 day-old *P. patens* cells transiently expressing (**a**) 2×35S::RFP-nonPTS1 and a PEX5 receptor with an unmodified C-terminal domain, whose expression is driven by a strong constitutive actin promoter; (**b**) 2×35S::RFP-PTS1 and a PEX5 receptor with an unmodified C-terminal domain, whose expression is driven by a strong constitutive actin promoter. In each panel, the left column shows fluorescence observed in the RFP channel, the middle column shows fluorescence in the GFP channel and the right column is the merge between of the RFP and GFP channels. Scale bar, 20 µm. Superimposed numbers represent the average localization score.

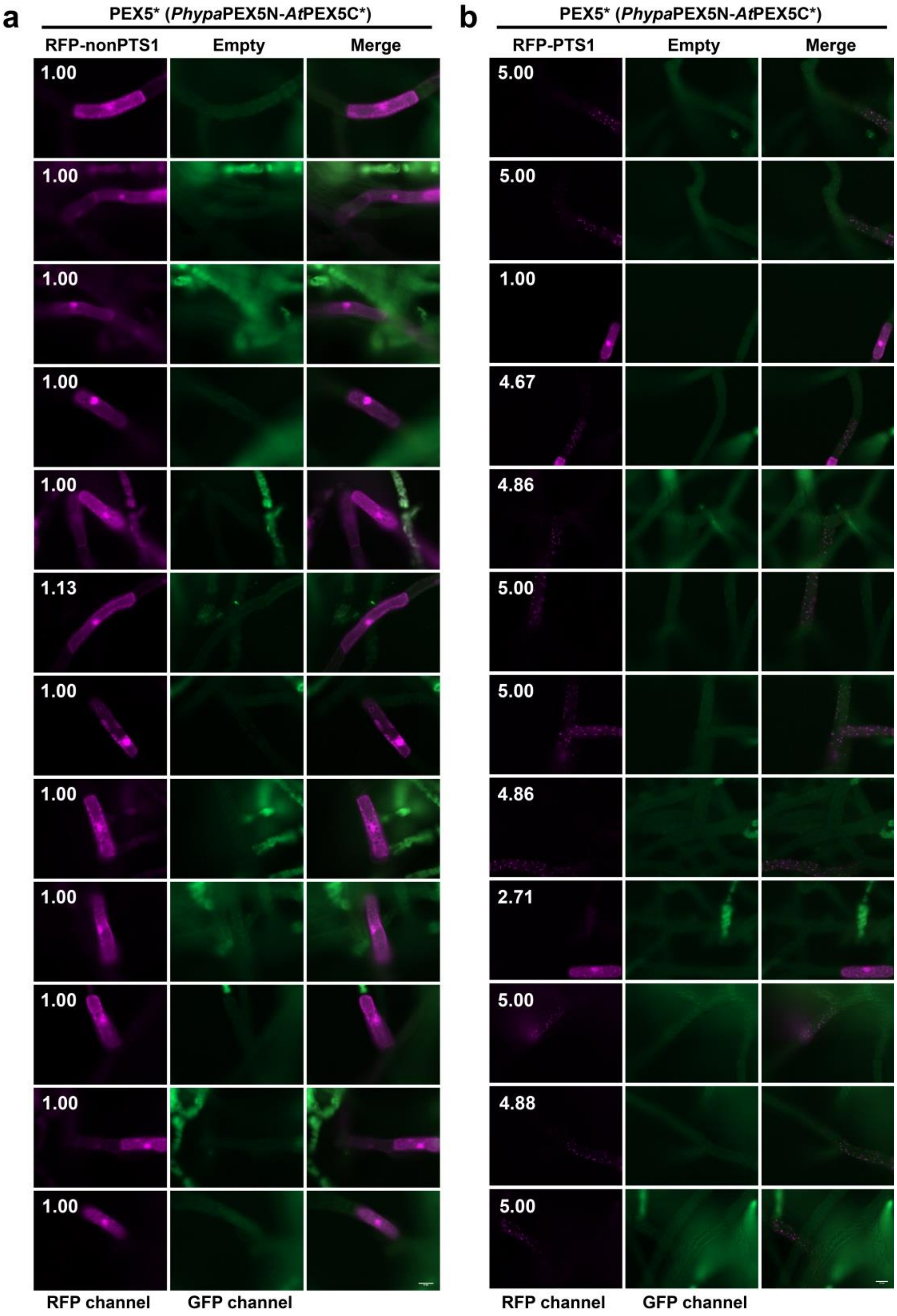

SupplementaryFigure 5 | 12 representative images of 6–7 day-old *P. patens* cells transiently expressing (**a**) 2×35S::RFP-nonPTS1 and a PEX5* receptor with the variant C-terminal domain, whose expression is driven by a strong constitutive actin promoter; (**b**) 2×35S::RFP-PTS1 and a PEX5* receptor with the variant C-terminal domain, whose expression is driven by a strong constitutive actin promoter. In each panel, the left column shows fluorescence observed in the RFP channel, the middle column shows fluorescence in the GFP channel and the right column is the merge between of the RFP and GFP channels. Scale bar, 20 µm.Superimposed numbers represent the average localization score.

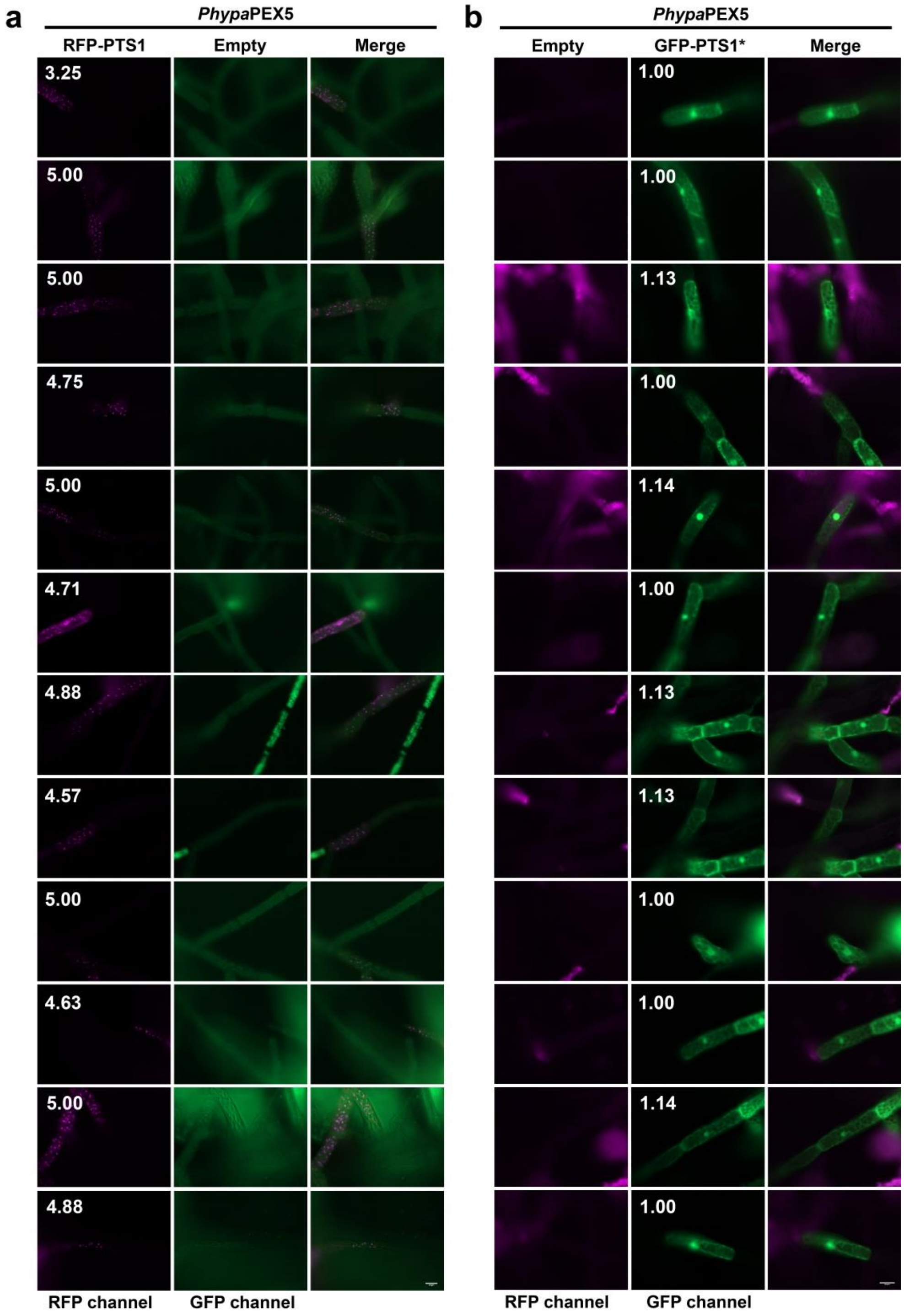

SupplementaryFigure 6 | 12 representative images of 6–7 day-old *P. patens* cells transiently expressing (**a**) 2×35S::RFP-PTS1 – only naturally occurring levels of endogenous *Phypa*PEX5 are present; (**b**) 2×35S::GFP-PTS1* – only naturally occurring levels of endogenous *Phypa*PEX5 are present. In each panel, the left column shows fluorescence observed in the RFP channel, the middle column shows fluorescence in the GFP channel and the right column is the merge between of the RFP and GFP channels. Scale bar, 20 µm. Superimposed numbers represent the average localization score.

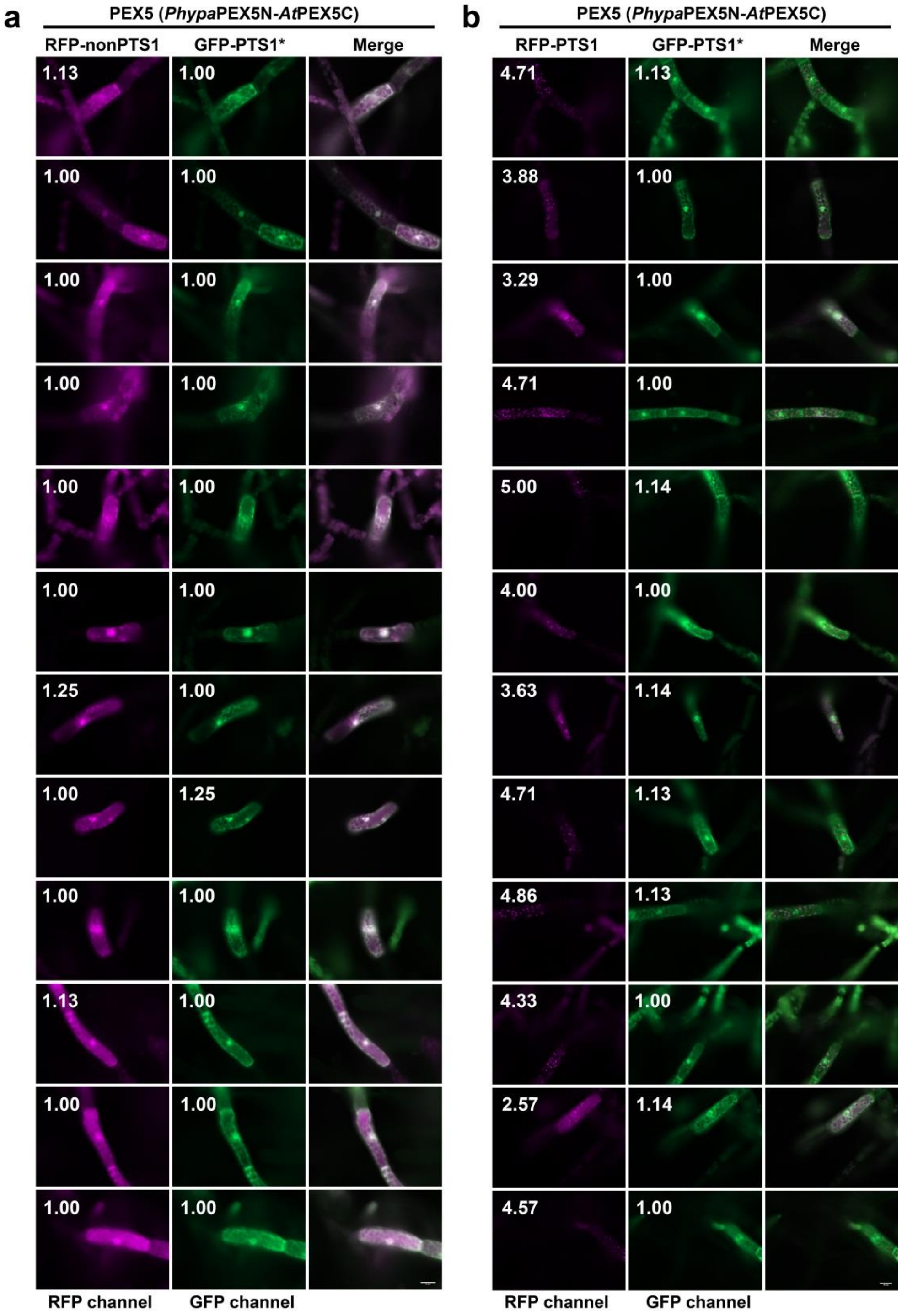

SupplementaryFigure 7 | 12 representative images of 6–7 day-old *P. patens* cells transiently co expressing (**a**) 2×35S::RFP-nonPTS1, 2×35S::GFP-PTS1* and the PEX5 receptor; (**b**) 2×35S::RFP-PTS1, 2×35S::GFP-PTS1* and the PEX5 receptor. In each panel, the left column shows fluorescence observed in the RFP channel, the middle column shows fluorescence in the GFP channel and the right column is the merge between of the RFP and GFP channels. Scale bar, 20 µm. Superimposed numbers represent the average localization score.

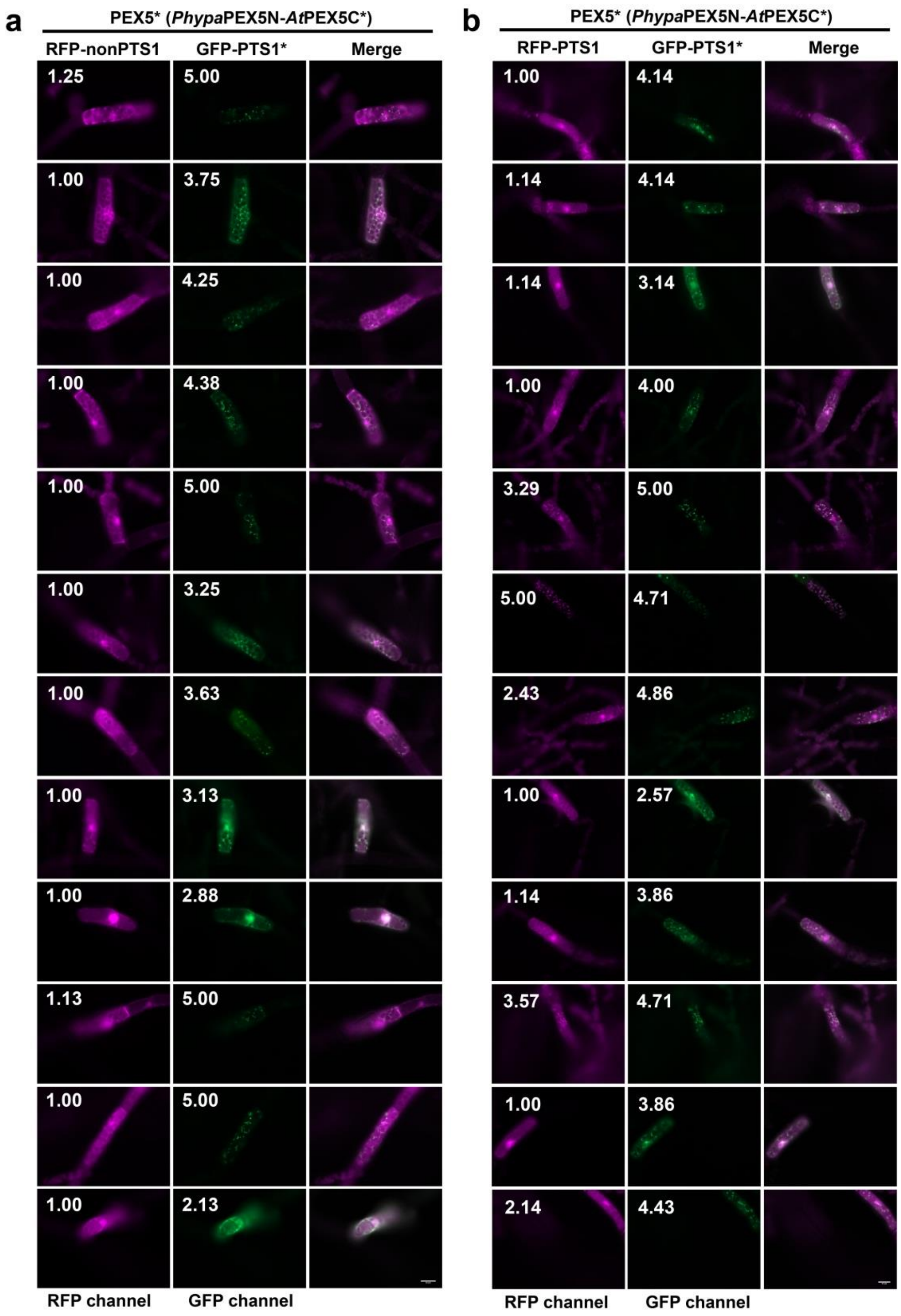

SupplementaryFigure 8 | 12 representative images of 6–7 day-old *P. patens* cells transiently co expressing (**a**) 2×35S::RFP-nonPTS1, 2×35S::GFP-PTS1* and the PEX5* receptor; (**b**) 2×35S::RFP-PTS1, 2×35S::GFP-PTS1* and the PEX5* receptor. In each panel, the left column shows fluorescence observed in the RFP channel, the middle column shows fluorescence in the GFP channel and the right column is the merge between of the RFP and GFP channels. Scale bar, 20 µm. Superimposed numbers represent the average localization score.

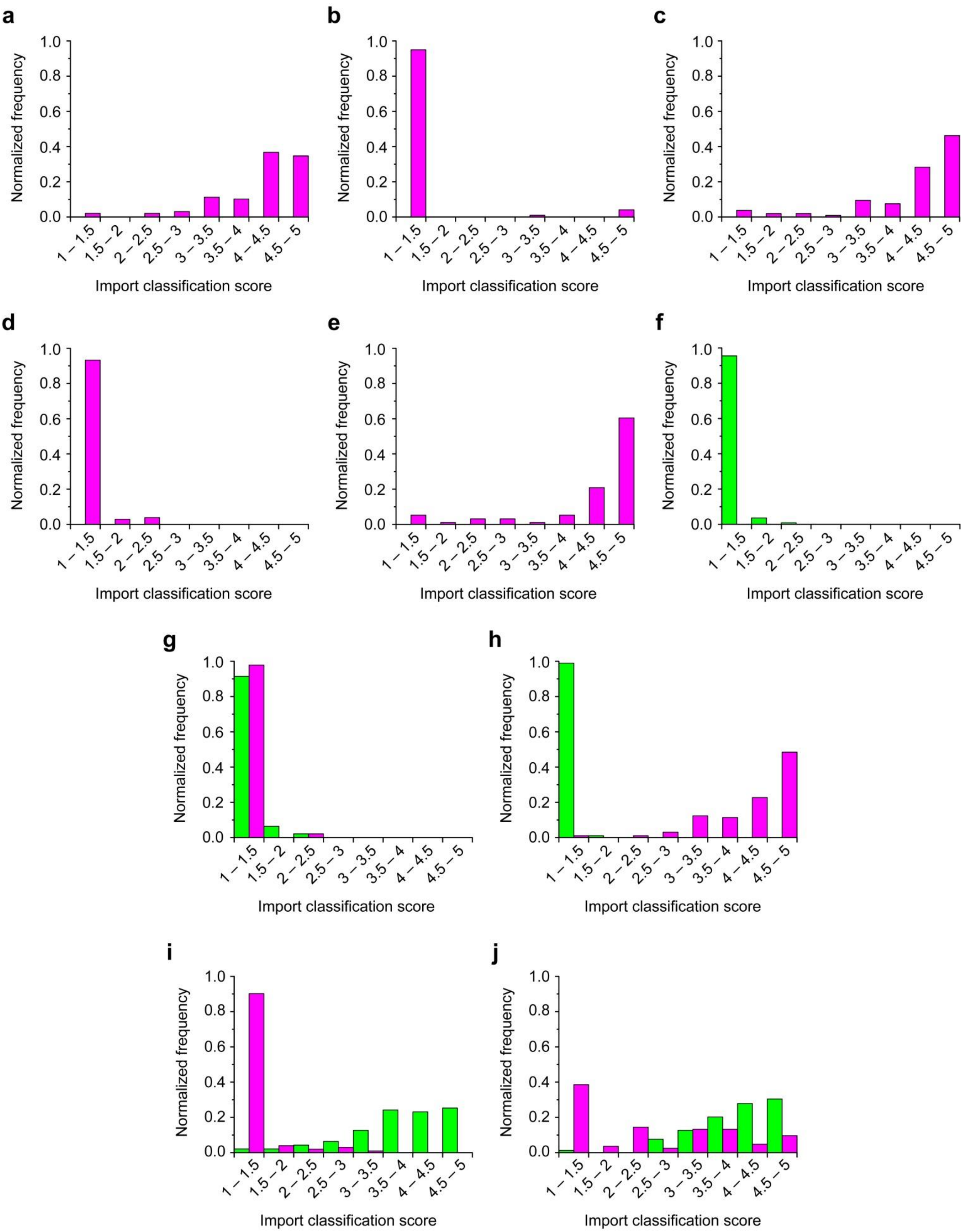

Supplementary Figure 9 | Histograms to show the normalized frequency of peroxisomal import classification scores for each experimental condition. (**a**) RFP-PTS1 only. (**b**) PEX5 & RFP-nonPTS1. (**c**) PEX5 & RFP-PTS1. (**d**) PEX5* & RFP-nonPTS1. (**e**) PEX5* & RFP-PTS1. (**f**) GFP-PTS1* only. (**g**) PEX5 & RFP-nonPTS1 + GFP-PTS1*. (**h**) PEX5 & RFP-PTS1 + GFP-PTS1*. (**i**) PEX5* & RFP-nonPTS1 + GFP-PTS1*. (**j**) PEX5* & RFP-PTS1 + GFP-PTS1*.

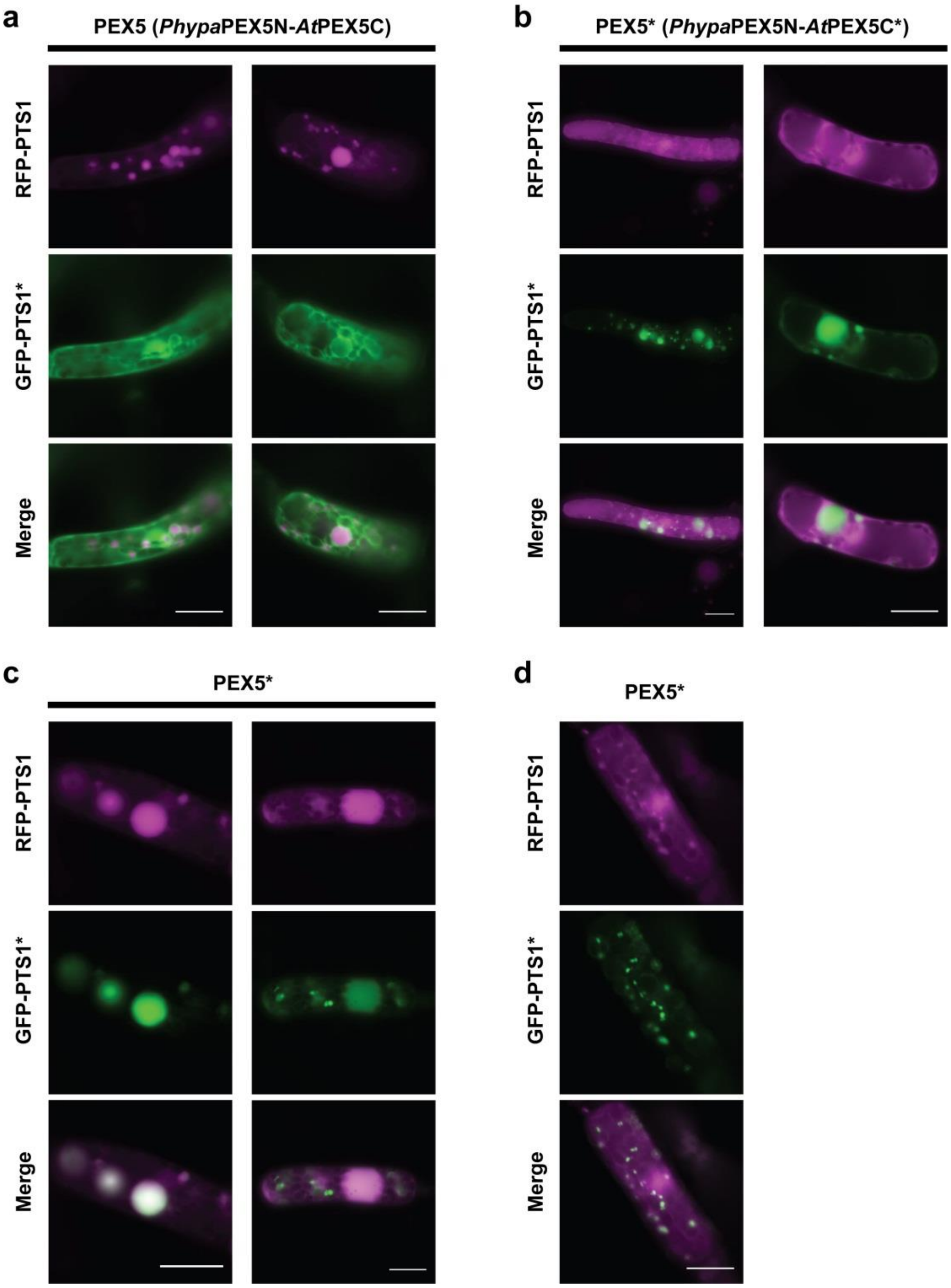

Supplementary Figure 10 | *In vivo* transient expression in a *P. patenspex11ko* mutant line, showing a complete switch in peroxisomal import and examples of co-localization in both the *Pppex11ko* mutant line and in wild-type *P. patens*. The phenotype of the *Pppex11ko* mutant line is larger peroxisomes which are fewer in number. (**a**) Images of 6–7 day-old *P. patens pex11ko* mutant cells transiently co-expressing2×35S::RFP-PTS1, 2×35S::GFP-PTS1* and the PEX5 receptor. (**b**) Images of 6–7 day-old *P. patenspex11ko* mutant cells transiently co-expressing 2×35S::RFP-PTS1, 2×35S::GFP-PTS1* and the PEX5* receptor.(**c**) Images of 6–7 day-old *P. patenspex11ko* mutant cells transiently co-expressing 2×35S::RFP-PTS1, 2×35S::GFP-PTS1* and the PEX5* receptor, in example cells where import of both fluorescent reporters is observed. (**d**) Images of 6–7 day-old *P. patens*wild-type cells transiently co-expressing 2×35S::RFP-PTS1, 2×35S::GFP-PTS1* and the PEX5* receptor, in an example cell where import of both fluorescent reporters is observed. Scale bar, 20 µm.

*At*Pex5C

ATGGGCAGCAGCCATCATCATCATCATCACAGCAGCGGCCTGGTGCCGCGCGGCAGCCATATGCAAGCTTCAGCCCCCGGGGAAT
GGGCTACTGAATATGAACAGCAGTATCTGGGGCCACCAAGTTGGGCTGATCAATTTGCAAATGAGAAACTTTCACATGGACCAGA
ACAGTGGGCTGATGAGTTTGCTTCCGGGAGAGGACAGCAAGAAACAGCTGAGGACCAATGGGTTAATGAGTTTTCAAAGTTGAAT
GTTGATGACTGGATAGATGAATTTGCTGAAGGTCCCGTGGGTGATAGTTCAGCTGATGCATGGGCAAATGCTTACGATGAGTTTC
TGAATGAGAAAAATGCTGGAAAACAAACCAGTGGTGTCTACGTCTTCTCTGACATGAATCCTTATGTGGGTCACCCTGAACCTAT
GAAAGAAGGGCAAGAATTGTTTCGAAAAGGACTTCTGAGTGAAGCAGCGCTTGCTCTAGAAGCTGAGGTTATGAAAAACCCTGAG
AATGCTGAAGGTTGGAGATTACTTGGGGTCACACACGCAGAGAACGATGATGATCAACAGGCAATAGCTGCAATGATGCGTGCAC
AGGAGGCTGATCCCACAAATCTAGAGGTGCTTCTTGCGCTTGGTGTGAGTCATACCAACGAGTTAGAGCAAGCAACTGCTTTGAA
ATATCTATATGGATGGCTGCGAAATCACCCAAAGTATGGAGCAATTGCGCCTCCGGAGCTAGCGGATTCTTTGTACCATGCTGAT
ATTGCTAGATTATTCAATGAAGCTTCTCAGTTGAATCCTGAGGACGCCGATGTGCATATAGTGTTGGGCGTGCTCTACAATCTGT
CGAGAGAGTTCGATAGAGCAATCACATCCTTCCAAACAGCATTACAACTAAAACCAAACGATTATTCTCTGTGGAATAAGCTAGG
TGCAACGCAAGCCAACAGTGTCCAGAGTGCTGATGCCATATCTGCTTATCAACAGGCTCTAGATTTAAAACCAAATTATGTTCGT
GCTTGGGCAAACATGGGAATCAGTTACGCAAACCAGGGGATGTACAAAGAATCAATCCCGTATTATGTCCGTGCCCTTGCGATGA
ATCCTAAAGCTGATAACGCATGGCAATACTTGAGACTCTCGTTAAGTTGTGCATCAAGGCAAGACATGATAGAAGCTTGTGAGTC
AAGGAATCTCGATCTCTTGCAGAAAGAATTCCCGCTGTGA

Supplementary Figure 11 | DNA sequence of *At*Pex5C.

*At*PEX5C

GSSHHHHHHSSGLVPRGSHMQASAPGEWATEYEQQYLGPPSWADQFANEKLSHGPEQWADEFASGRGQQETAEDQWVNEFSKLNV
DDWIDEFAEGPVGDSSADAWANAYDEFLNEKNAGKQTSGVYVFSDMNPYVGHPEPMKEGQELFRKGLLSEAALALEAEVMKNPEN
AEGWRLLGVTHAENDDDQQAIAAMMRAQEADPTNLEVLLALGVSHTNELEQATALKYLYGWLRNHPKYGAIAPPELADSLYHADI
ARLFNEASQLNPEDADVHIVLGVLYNLSREFDRAITSFQTALQLKPNDYSLWNKLGATQANSVQSADAISAYQQALDLKPNYVRA
WANMGISYANQGMYKESIPYYVRALAMNPKADNAWQYLRLSLSCASRQDMIEACESRNLDLLQKEFPL

Supplementary Figure 12 | Protein sequence of *At*PEX5C.

*At*Pex5C*(D505H-D507T-N601A mutant)

ATGGGCAGCAGCCATCATCATCATCATCACAGCAGCGGCCTGGTGCCGCGCGGCAGCCATATGCAAGCTTCAGCCCCCGGGGAAT
GGGCTACTGAATATGAACAGCAGTATCTGGGGCCACCAAGTTGGGCTGATCAATTTGCAAATGAGAAACTTTCACATGGACCAGA
ACAGTGGGCTGATGAGTTTGCTTCCGGGAGAGGACAGCAAGAAACAGCTGAGGACCAATGGGTTAATGAGTTTTCAAAGTTGAAT
GTTGATGACTGGATAGATGAATTTGCTGAAGGTCCCGTGGGTGATAGTTCAGCTGATGCATGGGCAAATGCTTACGATGAGTTTC
TGAATGAGAAAAATGCTGGAAAACAAACCAGTGGTGTCTACGTCTTCTCTGACATGAATCCTTATGTGGGTCACCCTGAACCTAT
GAAAGAAGGGCAAGAATTGTTTCGAAAAGGACTTCTGAGTGAAGCAGCGCTTGCTCTAGAAGCTGAGGTTATGAAAAACCCTGAG
AATGCTGAAGGTTGGAGATTACTTGGGGTCACACACGCAGAGAACCATGATACTCAACAGGCAATAGCTGCAATGATGCGTGCAC
AGGAGGCTGATCCCACAAATCTAGAGGTGCTTCTTGCGCTTGGTGTGAGTCATACCAACGAGTTAGAGCAAGCAACTGCTTTGAA
ATATCTATATGGATGGCTGCGAAATCACCCAAAGTATGGAGCAATTGCGCCTCCGGAGCTAGCGGATTCTTTGTACCATGCTGAT
ATTGCTAGATTATTCAATGAAGCTTCTCAGTTGAATCCTGAGGACGCCGATGTGCATATAGTGTTGGGCGTGCTCTACGCTCTGT
CGAGAGAGTTCGATAGAGCAATCACATCCTTCCAAACAGCATTACAACTAAAACCAAACGATTATTCTCTGTGGAATAAGCTAGG
TGCAACGCAAGCCAACAGTGTCCAGAGTGCTGATGCCATATCTGCTTATCAACAGGCTCTAGATTTAAAACCAAATTATGTTCGT
GCTTGGGCAAACATGGGAATCAGTTACGCAAACCAGGGGATGTACAAAGAATCAATCCCGTATTATGTCCGTGCCCTTGCGATGA
ATCCTAAAGCTGATAACGCATGGCAATACTTGAGACTCTCGTTAAGTTGTGCATCAAGGCAAGACATGATAGAAGCTTGTGAGTC
AAGGAATCTCGATCTCTTGCAGAAAGAATTCCCGCTGTGA

Supplementary Figure 13 | DNA sequence of *At*Pex5C*.(D505H-D507T-N601A mutant), mutations highlighted in grey.

*At*PEX5C*(D505H-D507T-N601A variant)

GSSHHHHHHSSGLVPRGSHMQASAPGEWATEYEQQYLGPPSWADQFANEKLSHGPEQWADEFASGRGQQETAEDQWVNEFSKLNV
DDWIDEFAEGPVGDSSADAWANAYDEFLNEKNAGKQTSGVYVFSDMNPYVGHPEPMKEGQELFRKGLLSEAALALEAEVMKNPEN
AEGWRLLGVTHAENHDTQQAIAAMMRAQEADPTNLEVLLALGVSHTNELEQATALKYLYGWLRNHPKYGAIAPPELADSLYHADI
ARLFNEASQLNPEDADVHIVLGVLYALSREFDRAITSFQTALQLKPNDYSLWNKLGATQANSVQSADAISAYQQALDLKPNYVRA
WANMGISYANQGMYKESIPYYVRALAMNPKADNAWQYLRLSLSCASRQDMIEACESRNLDLLQKEFPL

Supplementary Figure 14 | Protein sequence of *At*PEX5C*.(D505H-D507T-N601A variant), mutations highlighted in grey.

Pex5(*Phypa*Pex5N–*At*Pex5C)

ATGGCGTTTCGTGACTTGGTGATGGGAGGCGCCGGGTGTGCAGTCCCCGGGCAAGATGGCGCGTCGTCTTCGAATCCCTTGGGTG
GCTTGGCTGACTCGATCATTGGCTCTGCATCTAAAACTCAGGAGAGGATCCGGGAGATGCCAGGATTAGCTGGAGCAAGCCAAGC
TGGACCTCAATTTGGCCGAAATGGACCGCTTACCTCGCTTCCTGGCTCTGAATTTAATCAAGCCCAATGGCATGATGGGCAGGTG
CCGGAGTACATGCGAGGATTTCAGAGTGCGGACCCAAGAGAATTCACAGATGCTTGGAATCAGTCTGTGGAAAGGTGGCCTCCTC
CCCCACAGTTATCAACGGTTCCCGGAAATCAGGAAGGAGCGTTATTCTCAGAATTTGATAGTATATACGGGCAACAAGCTGGTCC
ATCCGGTGCGCCGTTGTTAGATGCCCCACCACAGCGTGTGTTGAATAATTTTCTTCATTCTTTCTTCGACAGCAGTCGAACGAAT
GCTCCTTTTCCTGCTGTTAGGCTGCCAGAGCTTGGACTTTCTCAGGCTGACAAGCGTCGGATAAGGGACCGTAGTCACATCATGG
GACGCCACATATTTGCTGACAAGGGCGATACTTTTGTTGATTCTCAGGTCAATGGTCTTCTACATTCCTTGGATATTGATGAAAG
TTTGGGAGCCCAAACTCGTGGTCCACTTGCTGGTCAGATTCCAGAATTTGAGCAATTGTGGAAAGAGGGTTTTGCTCAGAGTAAT
ATGCAAGTTGTGCCAAGACCTCCTATGTCACTTGGAACTCAGTGGGCAAATGAATTTCACAGCCAAGTTGGGCCTTCTGGGGCTC
CTCAAGGCTGGGCAGATGAGTTTGACAATCTACAGACTGGCAATAATTGGGCCAATCAGATGCAAGAGCAGCAAAGTCTGAGTCA
TCTTCGTAACGGGCAAATGGGCAATATGGCTGGGATGGAGCAAACTCGTTCACTTGTTCAGACTTTGTCTCAAAACCAGGACCCA
AAATTCCAGAGTTCCAAATTTTTGCAGTTTGTATCGAAAATGAGTCGTGGAGAACTTATCGTGGAAGATAATCAGTTCAAGCAAG
CTTCAGCCCCCGGGGAATGGGCTACTGAATATGAACAGCAGTATCTGGGGCCACCAAGTTGGGCTGATCAATTTGCAAATGAGAA
ACTTTCACATGGACCAGAACAGTGGGCTGATGAGTTTGCTTCCGGGAGAGGACAGCAAGAAACAGCCGAGGACCAATGGGTTAAT
GAGTTTTCAAAGTTGAATGTTGATGACTGGATAGATGAATTTGCTGAAGGTCCCGTGGGTGATAGTTCAGCTGATGCATGGGCAA
ATGCTTACGATGAGTTTCTGAATGAGAAAAATGCTGGAAAACAAACCAGTGGTGTCTACGTCTTCTCTGACATGAATCCTTATGT
GGGTCACCCTGAACCTATGAAAGAAGGGCAAGAATTGTTTCGAAAAGGACTTCTGAGTGAAGCAGCGCTTGCTCTAGAAGCTGAG
GTTATGAAAAACCCTGAGAATGCTGAAGGTTGGAGATTACTTGGGGTCACACACGCAGAGAACGATGATGATCAACAGGCAATAG
CTGCAATGATGCGTGCACAGGAGGCTGATCCCACAAATCTAGAGGTGCTTCTTGCGCTTGGTGTGAGTCATACCAACGAGTTAGA
GCAAGCAACTGCTTTGAAATATCTATATGGATGGCTGCGAAATCACCCAAAGTATGGAGCAATTGCGCCTCCGGAGCTAGCGGAT
TCTTTGTACCATGCTGATATTGCTAGATTATTCAATGAAGCTTCTCAGTTGAATCCTGAGGACGCCGATGTGCATATAGTGTTGG
GCGTGCTCTACAATCTGTCGAGAGAGTTCGATAGAGCAATCACATCCTTCCAAACAGCATTACAACTAAAACCAAACGATTATTC
TCTGTGGAATAAGCTAGGTGCAACGCAAGCCAACAGTGTCCAGAGTGCTGATGCCATATCTGCTTATCAACAGGCTCTAGATTTA
AAACCAAATTATGTTCGTGCTTGGGCAAACATGGGAATCAGTTACGCAAACCAGGGGATGTACAAAGAATCAATCCCGTATTATG
TCCGTGCCCTTGCGATGAATCCTAAAGCTGATAACGCATGGCAATACTTGAGACTCTCGTTAAGTTGTGCATCAAGGCAAGACAT
GATAGAAGCTTGTGAGTCAAGGAATCTCGATCTCTTGCAGAAAGAATTCCCGCTGTGA

Supplementary Figure 15 | DNA sequence of Pex5(*Phypa*Pex5N–*At*Pex5C). Grey sequence originates from*Phypa*Pex5N; yellow sequence originates from*At*Pex5C.

PEX5(*Phypa*PEX5N–*At*PEX5C)

MAFRDLVMGGAGCAVPGQDGASSSNPLGGLADSIIGSASKTQERIREMPGLAGASQAGPQFGRNGPLTSLPGSEFNQAQWHDGQV
PEYMRGFQSADPREFTDAWNQSVERWPPPPQLSTVPGNQEGALFSEFDSIYGQQAGPSGAPLLDAPPQRVLNNFLHSFFDSSRTN
APFPAVRLPELGLSQADKRRIRDRSHIMGRHIFADKGDTFVDSQVNGLLHSLDIDESLGAQTRGPLAGQIPEFEQLWKEGFAQSN
MQVVPRPPMSLGTQWANEFHSQVGPSGAPQGWADEFDNLQTGNNWANQMQEQQSLSHLRNGQMGNMAGMEQTRSLVQTLSQNQDP
KFQSSKFLQFVSKMSRGELIVEDNQVKQASAPGEWATEYEQQYLGPPSWADQFANEKLSHGPEQWADEFASGRGQQETAEDQWVN
EFSKLNVDDWIDEFAEGPVGDSSADAWANAYDEFLNEKNAGKQTSGVYVFSDMNPYVGHPEPMKEGQELFRKGLLSEAALALEAE
VMKNPENAEGWRLLGVTHAENDDDQQAIAAMMRAQEADPTNLEVLLALGVSHTNELEQATALKYLYGWLRNHPKYGAIAPPELAD
SLYHADIARLFNEASQLNPEDADVHIVLGVLYNLSREFDRAITSFQTALQLKPNDYSLWNKLGATQANSVQSADAISAYQQALDL
KPNYVRAWANMGISYANQGMYKESIPYYVRALAMNPKADNAWQYLRLSLSCASRQDMIEACESRNLDLLQKEFPL

Supplementary Figure 16 | Protein sequence of PEX5(*Phypa*PEX5N–*At*PEX5C). Grey sequence originates from*Phypa*PEX5N; yellow sequence originates from*At*PEX5C.

Pex5*(*Phypa*Pex5N–*At*Pex5C*)

ATGGCGTTTCGTGACTTGGTGATGGGAGGCGCCGGGTGTGCAGTCCCCGGGCAAGATGGCGCGTCGTCTTCGAATCCCTTGGGTG
GCTTGGCTGACTCGATCATTGGCTCTGCATCTAAAACTCAGGAGAGGATCCGGGAGATGCCAGGATTAGCTGGAGCAAGCCAAGC
TGGACCTCAATTTGGCCGAAATGGACCGCTTACCTCGCTTCCTGGCTCTGAATTTAATCAAGCCCAATGGCATGATGGGCAGGTG
CCGGAGTACATGCGAGGATTTCAGAGTGCGGACCCAAGAGAATTCACAGATGCTTGGAATCAGTCTGTGGAAAGGTGGCCTCCTC
CCCCACAGTTATCAACGGTTCCCGGAAATCAGGAAGGAGCGTTATTCTCAGAATTTGATAGTATATACGGGCAACAAGCTGGTCC
ATCCGGTGCGCCGTTGTTAGATGCCCCACCACAGCGTGTGTTGAATAATTTTCTTCATTCTTTCTTCGACAGCAGTCGAACGAAT
GCTCCTTTTCCTGCTGTTAGGCTGCCAGAGCTTGGACTTTCTCAGGCTGACAAGCGTCGGATAAGGGACCGTAGTCACATCATGG
GACGCCACATATTTGCTGACAAGGGCGATACTTTTGTTGATTCTCAGGTCAATGGTCTTCTACATTCCTTGGATATTGATGAAAG
TTTGGGAGCCCAAACTCGTGGTCCACTTGCTGGTCAGATTCCAGAATTTGAGCAATTGTGGAAAGAGGGTTTTGCTCAGAGTAAT
ATGCAAGTTGTGCCAAGACCTCCTATGTCACTTGGAACTCAGTGGGCAAATGAATTTCACAGCCAAGTTGGGCCTTCTGGGGCTC
CTCAAGGCTGGGCAGATGAGTTTGACAATCTACAGACTGGCAATAATTGGGCCAATCAGATGCAAGAGCAGCAAAGTCTGAGTCA
TCTTCGTAACGGGCAAATGGGCAATATGGCTGGGATGGAGCAAACTCGTTCACTTGTTCAGACTTTGTCTCAAAACCAGGACCCA
AAATTCCAGAGTTCCAAATTTTTGCAGTTTGTATCGAAAATGAGTCGTGGAGAACTTATCGTGGAAGATAATCAGTTCAAGCTTC
AGCCCCCGGGGAATGGGCTACTGAATATGAACAGCAGTATCTGGGGCCACCAAGTTGGGCTGATCAATTTGCAAATGAGAAACTT
TCACATGGACCAGAACAGTGGGCTGATGAGTTTGCTTCCGGGAGAGGACAGCAAGAAACAGCTGAGGACCAATGGGTTAATGAGT
TTTCAAAGTTGAATGTTGATGACTGGATAGATGAATTTGCTGAAGGTCCCGTGGGTGATAGTTCAGCTGATGCATGGGCAAATGC
TTACGATGAGTTTCTGAATGAGAAAAATGCTGGAAAACAAACCAGTGGTGTCTACGTCTTCTCTGACATGAATCCTTATGTGGGT
CACCCTGAACCTATGAAAGAAGGGCAAGAATTGTTTCGAAAAGGACTTCTGAGTGAAGCAGCGCTTGCTCTAGAAGCTGAGGTTA
TGAAAAACCCTGAGAATGCTGAAGGTTGGAGATTACTTGGGGTCACACACGCAGAGAACCATGATACTCAACAGGCAATAGCTGC
AATGATGCGTGCACAGGAGGCTGATCCCACAAATCTAGAGGTGCTTCTTGCGCTTGGTGTGAGTCATACCAACGAGTTAGAGCAA
GCAACTGCTTTGAAATATCTATATGGATGGCTGCGAAATCACCCAAAGTATGGAGCAATTGCGCCTCCGGAGCTAGCGGATTCTT
TGTACCATGCTGATATTGCTAGATTATTCAATGAAGCTTCTCAGTTGAATCCTGAGGACGCCGATGTGCATATAGTGTTGGGCGT
GCTCTACGCTCTGTCGAGAGAGTTCGATAGAGCAATCACATCCTTCCAAACAGCATTACAACTAAAACCAAACGATTATTCTCTG
TGGAATAAGCTAGGTGCAACGCAAGCCAACAGTGTCCAGAGTGCTGATGCCATATCTGCTTATCAACAGGCTCTAGATTTAAAAC
CAAATTATGTTCGTGCTTGGGCAAACATGGGAATCAGTTACGCAAACCAGGGGATGTACAAAGAATCAATCCCGTATTATGTCCG
TGCCCTTGCGATGAATCCTAAAGCTGATAACGCATGGCAATACTTGAGACTCTCGTTAAGTTGTGCATCAAGGCAAGACATGATA
GAAGCTTGTGAGTCAAGGAATCTCGATCTCTTGCAGAAAGAATTCCCGCTGTGA

Supplementary Figure 17 | DNA sequence of Pex5*(*Phypa*Pex5N–*At*Pex5C*). Grey sequence originates from*Phypa*Pex5N; yellow sequence originates from*At*Pex5C*.

PEX5*(*Phypa*PEX5N–*At*PEX5C*)

MAFRDLVMGGAGCAVPGQDGASSSNPLGGLADSIIGSASKTQERIREMPGLAGASQAGPQFGRNGPLTSLPGSEFNQAQWHDGQV
PEYMRGFQSADPREFTDAWNQSVERWPPPPQLSTVPGNQEGALFSEFDSIYGQQAGPSGAPLLDAPPQRVLNNFLHSFFDSSRTN
APFPAVRLPELGLSQADKRRIRDRSHIMGRHIFADKGDTFVDSQVNGLLHSLDIDESLGAQTRGPLAGQIPEFEQLWKEGFAQSN
MQVVPRPPMSLGTQWANEFHSQVGPSGAPQGWADEFDNLQTGNNWANQMQEQQSLSHLRNGQMGNMAGMEQTRSLVQTLSQNQDP
KFQSSKFLQFVSKMSRGELIVEDNQVKQASAPGEWATEYEQQYLGPPSWADQFANEKLSHGPEQWADEFASGRGQQETAEDQWVN
EFSKLNVDDWIDEFAEGPVGDSSADAWANAYDEFLNEKNAGKQTSGVYVFSDMNPYVGHPEPMKEGQELFRKGLLSEAALALEAE
VMKNPENAEGWRLLGVTHAENDDDQQAIAAMMRAQEADPTNLEVLLALGVSHTNELEQATALKYLYGWLRNHPKYGAIAPPELAD
SLYHADIARLFNEASQLNPEDADVHIVLGVLYNLSREFDRAITSFQTALQLKPNDYSLWNKLGATQANSVQSADAISAYQQALDL
KPNYVRAWANMGISYANQGMYKESIPYYVRALAMNPKADNAWQYLRLSLSCASRQDMIEACESRNLDLLQKEFPL

Supplementary Figure 18 | Protein sequence of PEX5*(*Phypa*PEX5N–*At*PEX5C*). Grey sequence originates from*Phypa*PEX5N; yellow sequence originates from*At*PEX5C*.

GFP-PTS1*

ATGGTGAGCAAGGGCGAGGAGCTGTTCACCGGGGTGGTGCCCATCCTGGTCGAGCTGGACGGCGACGTAAACGGCCACAAGTTCA
GCGTGTCCGGCGAGGGCGAGGGCGATGCCACCTACGGCAAGCTGACCCTGAAGTTCATCTGCACCACCGGCAAGCTGCCCGTGCC
CTGGCCCACCCTCGTGACCACCTTCACCTACGGCGTGCAGTGCTTCAGCCGCTACCCCGACCACATGAAGCAGCACGACTTCTTC
AAGTCCGCCATGCCCGAAGGCTACGTCCAGGAGCGCACCATCTTCTTCAAGGACGACGGCAACTACAAGACCCGCGCCGAGGTGA
AGTTCGAGGGCGACACCCTGGTGAACCGCATCGAGCTGAAGGGCATCGACTTCAAGGAGGACGGCAACATCCTGGGGCACAAGCT
GGAGTACAACTACAACAGCCACAACGTCTATATCATGGCCGACAAGCAGAAGAACGGCATCAAGGTGAACTTCAAGATCCGCCAC
AACATCGAGGACGGCAGCGTGCAGCTCGCCGACCACTACCAGCAGAACACCCCCATCGGCGACGGCCCCGTGCTGCTGCCCGACA
ACCACTACCTGAGCACCCAGTCCGCCCTGAGCAAAGACCCCAACGAGAAGCGCGATCACATGGTCCTGCTGGAGTTCGTGACCGC
CGCCGGGATCACTCACGGCATGGACGAGCTGTACAAGGATTGGTGGAGAGATCCTTATTCTCCTATGTATCAATCTTATTATTGA

Supplementary Figure 19 | DNA sequence of GFP-PTS1*.Green sequence represents GFP; grey sequence represents PTS1*.

GFP-PTS1*

MVSKGEELFTGVVPILVELDGDVNGHKFSVSGEGEGDATYGKLTLKFICTTGKLPVPWPTLVTTFTYGVQCFSRYPDHMKQHDFF
KSAMPEGYVQERTIFFKDDGNYKTRAEVKFEGDTLVNRIELKGIDFKEDGNILGHKLEYNYNSHNVYIMADKQKNGIKVNFKIRH
NIEDGSVQLADHYQQNTPIGDGPVLLPDNHYLSTQSALSKDPNEKRDHMVLLEFVTAAGITHGMDELYKDWWRDPYSPMYQSYY

Supplementary Figure 20 | Protein sequence of GFP-PTS1*. Green sequence represents GFP; grey sequence represents PTS1*.

RFP-PTS1

ATGGCCTCCTCCGAGGACGTCATCAAGGAGTTCATGCGCTTCAAGGTGCGCATGGAGGGCTCCGTGAACGGCCACGAGTTCGAGA
TCGAGGGCGAGGGCGAGGGCCGCCCCTACGAGGGCACCCAGACCGCCAAGCTGAAGGTGACCAAGGGCGGCCCCCTGCCCTTCGC
CTGGGACATCCTGTCCCCTCAGTTCCAGTACGGCTCCAAGGCCTACGTGAAGCACCCCGCCGACATCCCCGACTACTTGAAGCTG
TCCTTCCCCGAGGGCTTCAAGTGGGAGCGCGTGATGAACTTCGAGGACGGCGGCGTGGTGACCGTGACCCAGGACTCCTCCCTGC
AGGACGGCGAGTTCATCTACAAGGTGAAGCTGCGCGGCACCAACTTCCCCTCCGACGGCCCCGTAATGCAGAAGAAGACCATGGG
CTGGGAGGCCTCCACCGAGCGGATGTACCCCGAGGACGGCGCCCTGAAGGGCGAGATCAAGATGAGGCTGAAGCTGAAGGACGGC
GGCCACTACGACGCCGAGGTCAAGACCACCTACATGGCCAAGAAGCCCGTGCAGCTGCCCGGCGCCTACAAGACCGACATCAAGC
TGGACATCACCTCCCACAACGAGGACTACACCATCGTGGAACAGTACGAGCGCGCCGAGGGCCGCCACTCCACCGGCGCCGATGG
GGAGACTATAGTTGTTGCTGGTGGCATGAAATCTAGGTTATAG

Supplementary Figure 21 | DNA sequence of RFP-PTS1. Magenta sequence represents RFP; grey sequence represents PTS1.

RFP-PTS1

MASSEDVIKEFMRFKVRMEGSVNGHEFEIEGEGEGRPYEGTQTAKLKVTKGGPLPFAWDILSPQFQYGSKAYVKHPADIPDYLKL
SFPEGFKWERVMNFEDGGVVTVTQDSSLQDGEFIYKVKLRGTNFPSDGPVMQKKTMGWEASTERMYPEDGALKGEIKMRLKLKDG
GHYDAEVKTTYMAKKPVQLPGAYKTDIKLDITSHNEDYTIVEQYERAEGRHSTGADGETIVVAGGMKSRL

Supplementary Figure 22 | Protein sequence of RFP-PTS1. Magenta sequence represents RFP; grey sequence represents PTS1.

RFP-nonPTS1

ATGGCCTCCTCCGAGGACGTCATCAAGGAGTTCATGCGCTTCAAGGTGCGCATGGAGGGCTCCGTGAACGGCCACGAGTTCGAGA
TCGAGGGCGAGGGCGAGGGCCGCCCCTACGAGGGCACCCAGACCGCCAAGCTGAAGGTGACCAAGGGCGGCCCCCTGCCCTTCGC
CTGGGACATCCTGTCCCCTCAGTTCCAGTACGGCTCCAAGGCCTACGTGAAGCACCCCGCCGACATCCCCGACTACTTGAAGCTG
TCCTTCCCCGAGGGCTTCAAGTGGGAGCGCGTGATGAACTTCGAGGACGGCGGCGTGGTGACCGTGACCCAGGACTCCTCCCTGC
AGGACGGCGAGTTCATCTACAAGGTGAAGCTGCGCGGCACCAACTTCCCCTCCGACGGCCCCGTAATGCAGAAGAAGACCATGGG
CTGGGAGGCCTCCACCGAGCGGATGTACCCCGAGGACGGCGCCCTGAAGGGCGAGATCAAGATGAGGCTGAAGCTGAAGGACGGC
GGCCACTACGACGCCGAGGTCAAGACCACCTACATGGCCAAGAAGCCCGTGCAGCTGCCCGGCGCCTACAAGACCGACATCAAGC
TGGACATCACCTCCCACAACGAGGACTACACCATCGTGGAACAGTACGAGCGCGCCGAGGGCCGCCACTCCACCGGCGCCGATAT
TATTGCTGCTGTTGATGCTTCTTATAATTCTTCTACTCTTTGA

Supplementary Figure 23 | DNA sequence of RFP-nonPTS1. Magenta sequence represents RFP; grey sequence represents nonPTS1.

RFP-nonPTS1

MASSEDVIKEFMRFKVRMEGSVNGHEFEIEGEGEGRPYEGTQTAKLKVTKGGPLPFAWDILSPQFQYGSKAYVKHPADIPDYLKL
SFPEGFKWERVMNFEDGGVVTVTQDSSLQDGEFIYKVKLRGTNFPSDGPVMQKKTMGWEASTERMYPEDGALKGEIKMRLKLKDG
GHYDAEVKTTYMAKKPVQLPGAYKTDIKLDITSHNEDYTIVEQYERAEGRHSTGADIIAAVDASYNSSTL

Supplementary Figure 24 | Protein sequence of RFP-nonPTS1. Magenta sequence represents RFP; grey sequence represents nonPTS1.

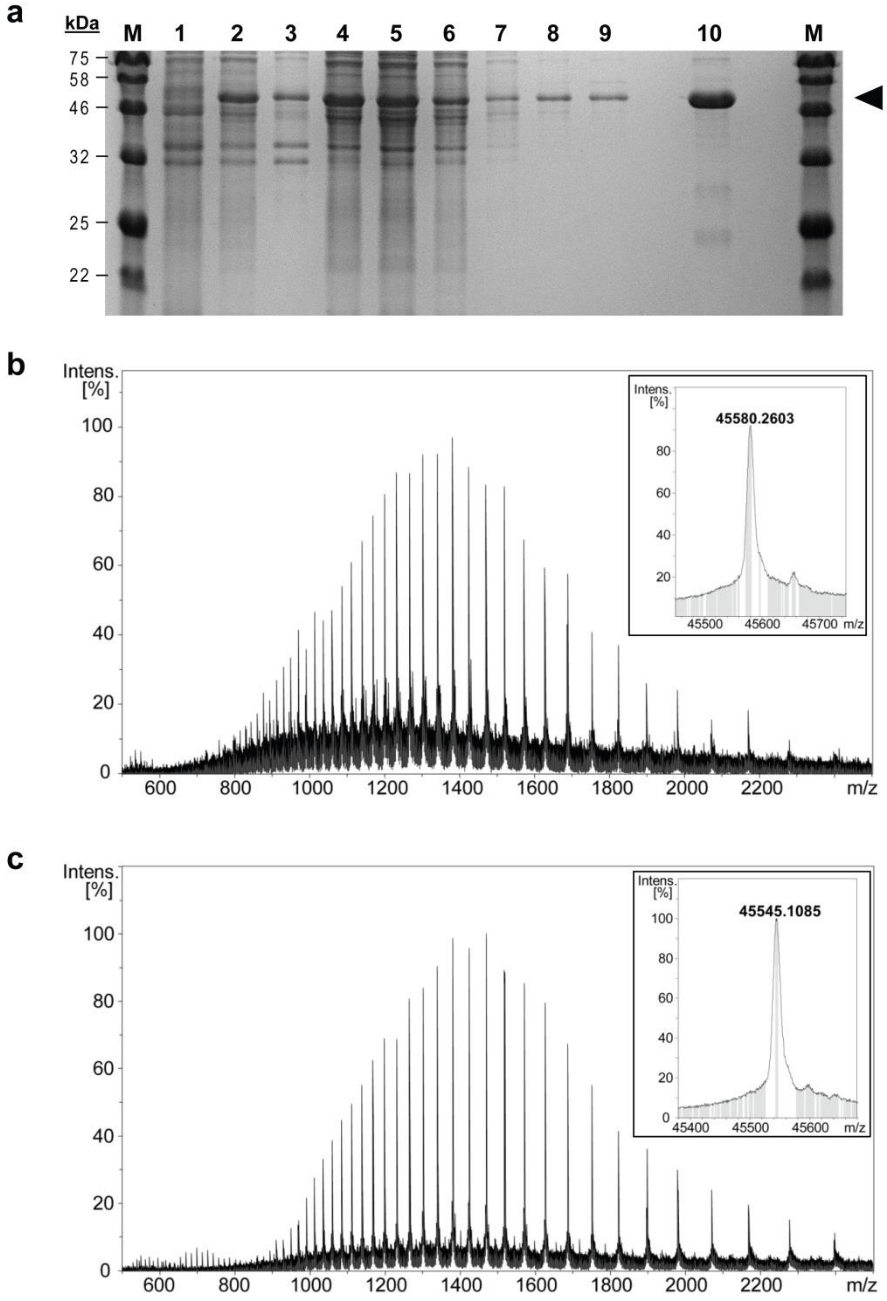

Supplementary Figure 25 | Characterization of purified protein. (**a**) Typical protein purification results: Coomassie blue-stained SDS-PAGE gel for *At*PEX5C. The band in lane 10 at approximately 50 kDa(indicated by filled arrowhead) represents *At*PEX5C. M, protein standard markers; 1, non-induced BL21-Gold (DE3) cells containing His_6-*At*PEX5C.pET-28b; 2, autoinduced BL21-Gold (DE3) cells containing His_6-*At*PEX5C.pET-28b; 3, pellet after cell lysis; 4, supernatant after cell lysis; 5, supernatant after incubation with cobalt-agarose resin; 6–9, buffer washes 1–4 of cobalt-agarose resin; 10, elution of protein using 200 mM imidazole. (**b**) Mass spectrum and deconvoluted mass data (inset) for wild-type *At*PEX5C. Expected mass: 45,580.3 Da. Observed mass: 45,580.3 Da. C) Mass spectrum and deconvoluted mass data (inset) for *At*PEX5C* (*At*PEX5C D505H-D507T-N601A). Expected mass: 45,545.3 Da. Observed mass: 45,545.1 Da.

Supplementary Tables

Supplementary Table 1| Summary of the changes in peptide binding seen with each *At*PEX5C variant. Proteins were grouped into four categories based on their apparent peptide-binding profile.

Group based on peptide-binding changes			
**1. Similar profile to wild-type *At*PEX5C**	**2. Background intensity of peptides pulled down**	**3. Only strong native PTS1s pulled down**	**4. Pull-down of non-PTS1 peptides seen**
D505A	D505K	V533A	D505H
D507A	D505K-D507K	V533W	T536W
D507K	N537A	T536N	N601A
T536A	N628A	N537T	D505H-N601A
E538A	R659A	N537Q	D505H-D507H-N601A
N601Q	N663A	F613A	D505H-D507T-N601A
Y647F	D505F-N601A	A632G	D505H-D507T-E538A-N601A
S667A	D505F-D507F-N601A	N636A	
D505H-T536W	D505H-N601A-N636A		
D505H-D507V-N601A			

Supplementary Table 2 | Results of image classifications following analysis of images collected after transfection with different plasmid combinations. A score of 5 for an individual image corresponded to 100% peroxisomal localization, while a score of 1 indicated complete cytosolic localization of fluorescence.

Experiment	Images	Average GFP localization classification	Average RFP localization classification
GFP-PTS1*	112	1.11 ± 0.02	-
PEX5 & RFP-nonPTS1	100	-	1.27 ± 0.09
PEX5 & RFP-PTS1	115	-	4.18 ± 0.08
PEX5* & RFP-nonPTS1	104	-	1.14 ± 0.03
PEX5* & RFP-PTS1	112	-	4.32 ± 0.09
RFP-PTS1 Only	109	-	4.17± 0.07
PEX5 & RFP-nonPTS1 + GFP- PTS1*	94	1.15 ± 0.02	1.08 ± 0.02
PEX5 & RFP-PTS1 + GFP- PTS1*	97	1.10 ± 0.01	4.23 ± 0.07
PEX5* & RFP-nonPTS1 + GFP- PTS1*	102	3.86 ± 0.09	1.19 ± 0.04
PEX5* & RFP-PTS1 + GFP- PTS1*	84	4.05 ± 0.08	2.48 ± 0.15

Supplementary Table 3 | P-values for statistical comparison of RFP histograms shown in Supplementary Figure 9 using a Kolmogorov-Smirnov Test. (n.s): not significant.

Experiment	PEX5 & RFP-nonPTS1	PEX5 & RFP-PTS1	PEX5* & RFP-nonPTS1	PEX5* & RFP-PTS1	RFP-PTS1 Only	PEX5 & RFP-nonPTS1 + GFP-PTS1*	PEX5 & RFP-PTS1 + GFP-PTS1*	PEX5* & RFP-nonPTS1 + GFP-PTS1*	PEX5* & RFP-PTS1 + GFP- PTS1*
PEX5 & RFP-nonPTS1	-	1.8×10^{-6}	1 (n.s.)	6.8×10^{-7}	1.8×10^{-6}	1 (n.s.)	2.2×10^{-6}	1 (n.s.)	2.1×10^{-13}
PEX5 & RFP-PTS1	-	-	3.4×10^{-6}	0.25 (n.s.)	0.48 (n.s.)	2.5×10^{-6}	1 (n.s.)	1.8×10^{-6}	1.9×10^{-14}
PEX5* & RFP-nonPTS1	-	-	-	1.2×10^{-6}	5.1×10^{-6}	1 (n.s.)	7.5×10^{-6}	1 (n.s.)	6.5×10^{-13}
PEX5* & RFP-PTS1	-	-	-	-	0.003	1.1×10^{-6}	0.46 (n.s)	4.7×10^{-7}	2.0×10^{-12}
RFP-PTS1 Only	-	-	-	-	-	2.9×10^{-6}	0.3 (n.s.)	1.8×10^{-6}	1.3×10^{-13}
PEX5 & RFP-nonPTS1 + GFP- PTS1*	-	-	-	-	-	-	4.4×10^{-6}	0.93 (n.s.)	2.0×10^{-14}
PEX5 & RFP-PTS1 + GFP-PTS1*	-	-	-	-	-	-	-	2.7×10^{-6}	2.0×10^{-13}
PEX5* & RFP-nonPTS1 + GFP- PTS1*	-	-	-	-	-	-	-	-	1.4×10^{-11}
PEX5* & RFP-PTS1 + GFP-PTS1*	-	-	-	-	-	-	-	-	-

Supplementary Table 4 | P-values for statistical comparison of GFP histograms shown in Supplementary Figure 9 using a Kolmogorov-Smirnov Test. (n.s): not significant.

Experiment	GFP-PTS1*	PEX5 & RFP-nonPTS1 + GFP-PTS1*	PEX5 & RFP-PTS1 + GFP-PTS1*	PEX5* & RFP-nonPTS1 + GFP-PTS1*	PEX5* & RFP-PTS1 + GFP-PTS1*
GFP-PTS1*	-	1 (n.s.)	1 (n.s.)	4.9×10^{-6}	3.8×10^{-6}
PEX5 & RFP-nonPTS1 + GFP- PTS1*	-	-	1 (n.s.)	1.4×10^{-6}	1.6×10^{-6}
PEX5 & RFP-PTS1 + GFP-PTS1*	-	-	-	3.6×10^{-6}	1.8×10^{-6}
PEX5* & RFP-nonPTS1 + GFP- PTS1*	-	-	-	-	0.78 (n.s.)
PEX5* & RFP-PTS1 + GFP-PTS1*	-	-	-	-	-
